# Patterns and changes in life expectancy in China, 1990-2016

**DOI:** 10.1371/journal.pone.0231007

**Published:** 2020-04-01

**Authors:** Hai Chen, Yun Qian, Yunqiu Dong, Zhijie Yang, Liangliang Guo, Jia Liu, Qian Shen, Lu Wang

**Affiliations:** Department of Chronic Disease Control, Center for Disease Control and Prevention, Wuxi, Jiangsu, China; Aarhus University and UCLA, UNITED STATES

## Abstract

**Objective:**

To achieve the goal of “healthy China 2030”, reasonable health policies must be developed based on the changes of death spectrum. We aim to investigate the temporal patterns of life expectancy (LE) and age/cause-specific contributions from 1990 to 2016.

**Methods:**

Joinpoint regression model was used with Arriaga’s decomposition method.

**Results:**

LE in China has reached to 76.3 years in 2016 with an increase of 9.44 years from 1990. From 1990 to 2002, a remarkable reduction in infant mortality accounted for an increase of 1.27 years (35.39%) to LE which mainly resulted from diarrhea, lower respiratory, and other common infectious diseases (1.00 years, 27.79%). After 2002, those aged 65+ years contributed most to increased LE and the most prominent causes included cardiovascular diseases (0.67 years, 23.36%), chronic respiratory diseases (0.54 years, 18.76%) and neoplasms (0.39 years, 13.44%). Moreover, the effects of transport injuries changed from negative to positive. After 2007, contributions of transport and unintentional injuries increased especially for males. And for females contributions of cardiovascular diseases sharply increased LE by 1.17 years (32.26%).

**Conclusion:**

More attention should be paid to cardiovascular diseases, chronic respiratory diseases and neoplasms which were mainly attributed to the increase of LE, especially for males and elderly population.

## Introduction

Life expectancy (LE) is a useful measure of population health due to its capacity for summarizing mortality in a single measure [1]. LE represents the average years a person is expected to live based on the overall mortality level of a population. It is an important index reflecting the social economy and healthcare development [2]. LE had increased steadily in most countries in the past 30 years including China, which has made extraordinary progress on LE from 60.9 years in 1970 to 76.3 in 2016 [3].

However, cause-specific death spectrum in China has changed due to changes in lifestyles and living conditions, along with the aging of the population and urbanization [4]. The gap in LE between two years is an algebraic function of underlying age and cause-specific mortality rates, which means that it can be partitioned into age and cause-specific components [5]. And decomposition methods are ideal for determining the positive and negative contributions of age and cause-specific mortality rates [6].

Previous studies [7, 8] usually used cause-eliminated life tables with potential gains in life expectancy as a research method to determine the impact of cause-specific death on life expectancy. However, this approach is only focused on a single population and the development of single decrement, while the decomposition method is more comparative and can assign responsibility for mortality variation to particular age groups or causes of death [9]. Moreover, similar studies in China have focused on data from short time-spans, and do not reflect trends of the nationwide impact [10, 11]. This study focuses on a 27-year time span and uses the nationwide data derived from global burden of disease study (GBD) which involves comprehensive sources of data from China and accurate methods for data processing and modelling.

The Chinese government has attached great importance to the improvement of the public health. A program of action to promote a healthier China named “healthy China 2030” was implemented in 2016. One of its goals is to increase the national LE to 79.0 by 2030 [12, 13]. To achieve this goal, reasonable health policies must be developed based on the changes in death spectrum. Therefore, the purpose of this study is to investigate the temporal patterns of LE and age/cause-specific contributions from 1990 to 2016 in China.

## Materials and methods

### Data sources

Mortality estimates for the period 1990–2016 in China, as tabulated in the causes of death, sex, age groups, were retrieved from global burden of disease study (GBD) 2016 [14]. The two primary sources of data for China are surveillance data from the China Disease Surveillance Points (DSP) system and vital registration data collected by the Chinese Center for Disease Control and Prevention (CDC) [15]. Causes of death were identified based on the 9th and 10th revision of the International Classification of Disease. The so-called garbage codes were redistributed using the method established by Naghavi et al [16]. Detailed methods and strategies for data standardisation and processing are outlined in S1 Appendix.

Causes of death are organised as a hierarchy (S1 Table), with each level composed of causes of death that are mutually exclusive and collectively exhaustive, including 7 subsets of communicable, maternal, neonatal, and nutritional diseases (CMNNs) (e.g. HIV/AIDS and tuberculosis, Diarrhea, Maternal disorders), 10 subsets of non-communicable diseases (NCDs) (e.g. neoplasms, cardiovascular diseases, chronic respiratory diseases) and 4 subsets of injuries (e.g. self-harm and interpersonal violence, transport injuries).

The analytical framework includes infant age group (<1 year) and 17 non-infant age groups starting with age 1–4 years, then proceeding in five-year age groups until the terminal age group of 85+. In some cases, results are reported for aggregate age intervals (1–14 years, 15–49 years, 50–64 years, and 65+ years) according to the grouping criteria from the Chinese CDC [17]. Number of deaths and population for each age group within the aggregate interval were added together. The total number of death was divided by the total number of population to obtain the mortality of each aggregate interval.

### Statistical analysis

LEs were calculated with standard abridged life tables, stratified by gender. Joinpoint regression model was used to investigate the temporal pattern of LE in China from 1990 to 2016, which is often used to describe changes in trend data with a few continuous linear segments [18]. The slope coefficients for each segment was calculated using linear model stratified by gender. Auto correlated errors were used to capture the unexplained component correlated over time [19]. In consideration of the results of Monte Carlo Permutation technique and unnecessary complications, the best model with locations of 2 joinpoints was selected [20].

Contributions to the absolute difference in LE of each age group and specific causes of death were estimated with the Arriaga’s decomposition method [21]. In our study there are two steps, the first a decomposition by age group and the second by cause of death within an age group [22]. Detailed procedure is described below.

Decomposing by each age group with an interval of *n*: *TE*_*x*_ is the total contribution between ages *x* and *x+n*, *l*_*x*_ is the number of individuals alive at age *x*, *l*_*0*_ is the cohort size at the start, *L*_*x*_ is the number of person-years lived between ages *x* and *x+n*, and *T*_*x+n*_ is the total number of person-years lived above age x+n. *l*_*x+n*_ is the number of individuals alive at age *x+n*. “1” represents the reference year while “2” represents the contrastive year.

$$TE_{x}=\left[\frac{l_{x}^{1}}{l_{0}}\times \left(\frac{L_{x}^{2}}{l_{x}^{2}}-\frac{L_{x}^{1}}{l_{x}^{1}}\right)\right]+\left[\frac{T_{x+n}^{2}}{l_{0}}\times \left(\frac{l_{x}^{1}}{l_{x}^{2}}-\frac{l_{x+n}^{1}}{l_{x+n}^{2}}\right)\right]$$ (1)

Decomposition by cause of death within an age group with an interval of *n*: $TE_{x}^{i}$ is the total contribution between ages *x* and *x+n* due to cause *i*, $R_{x}^{i}$ is the specific mortality rate between ages *x* and *x+n* due to cause *i*. *S*_*x*_ is the all-cause mortality rate between ages *x* and *x+n*. “1” represents the reference year while “2” represents the contrastive year.

$$TE_{x}^{i}=TE_{x}\times \left[\frac{R_{x}^{i,2}-R_{x}^{i,1}}{S_{x}^{2}-S_{x}^{1}}\right]$$ (2)

Joinpoint regression analyses were carried out using Joinpoint Regression Program, Version 4.6.0.0 (US National Cancer Institute, MD). Statistical analysis of decomposition was carried out with R version 3.4.1 (R Foundation for Statistical Computing, Vienna, Austria).

## Results

### Temporal patterns

**Fig 1:**
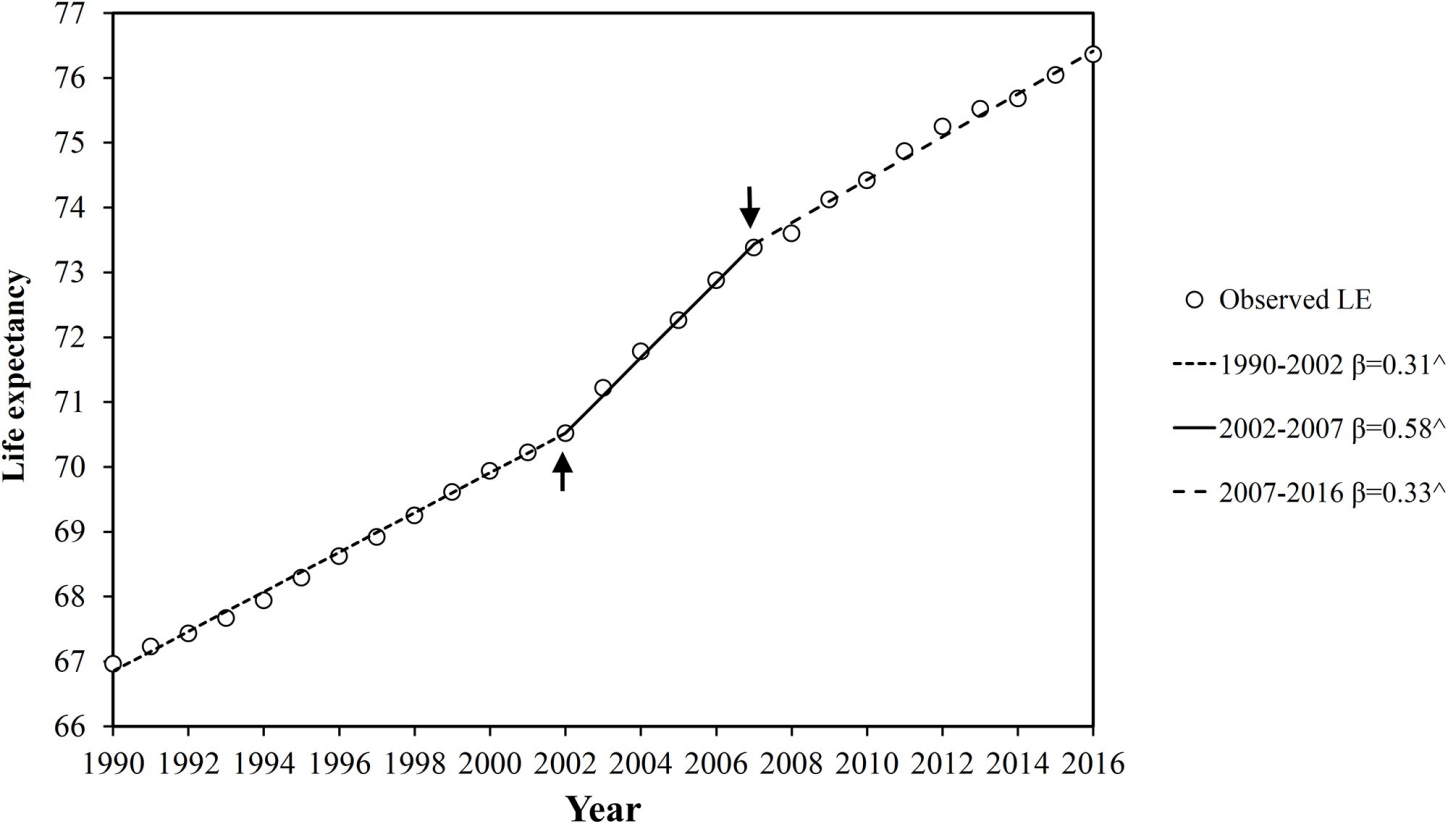
Temporal patterns in life expectancy in China from 1990 to 2016. Abbreviation: LE, life expectancy; β: slope coefficient; ^ Indicates that the slope coefficient is significantly different from zero at α = 0.05 level. Arrow indicate two joinpoints (2002 and 2007) for the trend of life expectancy from 1990 to 2016.

Between 1990 and 2016, LE in China increased annually with 2 joinpoints identified in 2002 and 2007 (Fig 1). LE increased significantly by 0.31 (95% CI: 0.29–0.32) per year from 1990 to 2002. And starting in 2002, a sharp increase was observed with 0.58 (95% CI: 0.52–0.65) per year until 2007 followed by a deceleration in increase (0.33 per year, 95% CI: 0.30–0.36) and reached to 76.3 years in 2016. Similar trends of LE for male and female were shown in S1 Fig with estimated regression coefficients and annual changes (S2 Table). As a result, LE gap of three segments was +3.58, +2.86 and +3.00 years, respectively (Table 1).

**Table 1:** Life expectancy and gaps for each segment.

Segments		Life expectancy		Life expectancy gap
1990	2002	66.88	70.46	+3.58
2002	2007	70.46	73.32	+2.86
2007	2016	73.32	76.33	+3.00

### Age-specific contributions

**Fig 2:**
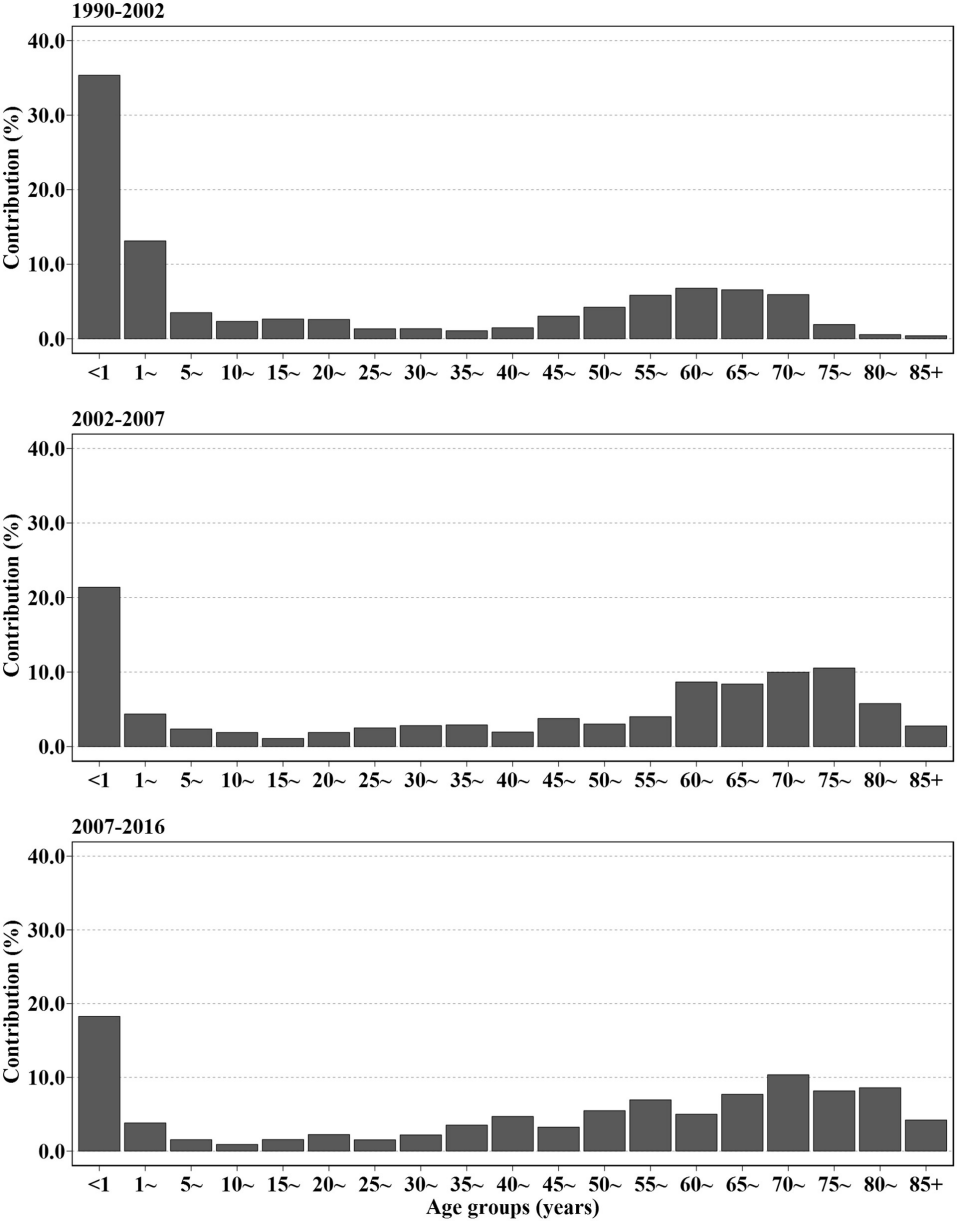
Age-specific contributions to life expectancy in different segments.

From 1990 to 2002, a remarkable reduction in infant mortality accounted for 1.27 years (35.39%) increase to LE (Fig 2). However, the impact of infant mortality rate was weakened with 0.61 years (21.43%) during 2002–2007 and 0.56 years (18.67%) during 2007–2016. The contribution of adolescent (aged 1–14 years) also decreased from 0.68 years (18.96%) to 0.19 years (6.42%). After 2002, the major group contributing to the increased LE were older adults (aged 65+ years) which accounted for 37.43%. And the contributions of young adults (aged 15–49 years) and middle-aged adults (aged 50–64 years) were relatively stable with a proportion of 15%~20%, respectively.

### Cause-specific contributions

**Fig 3:**
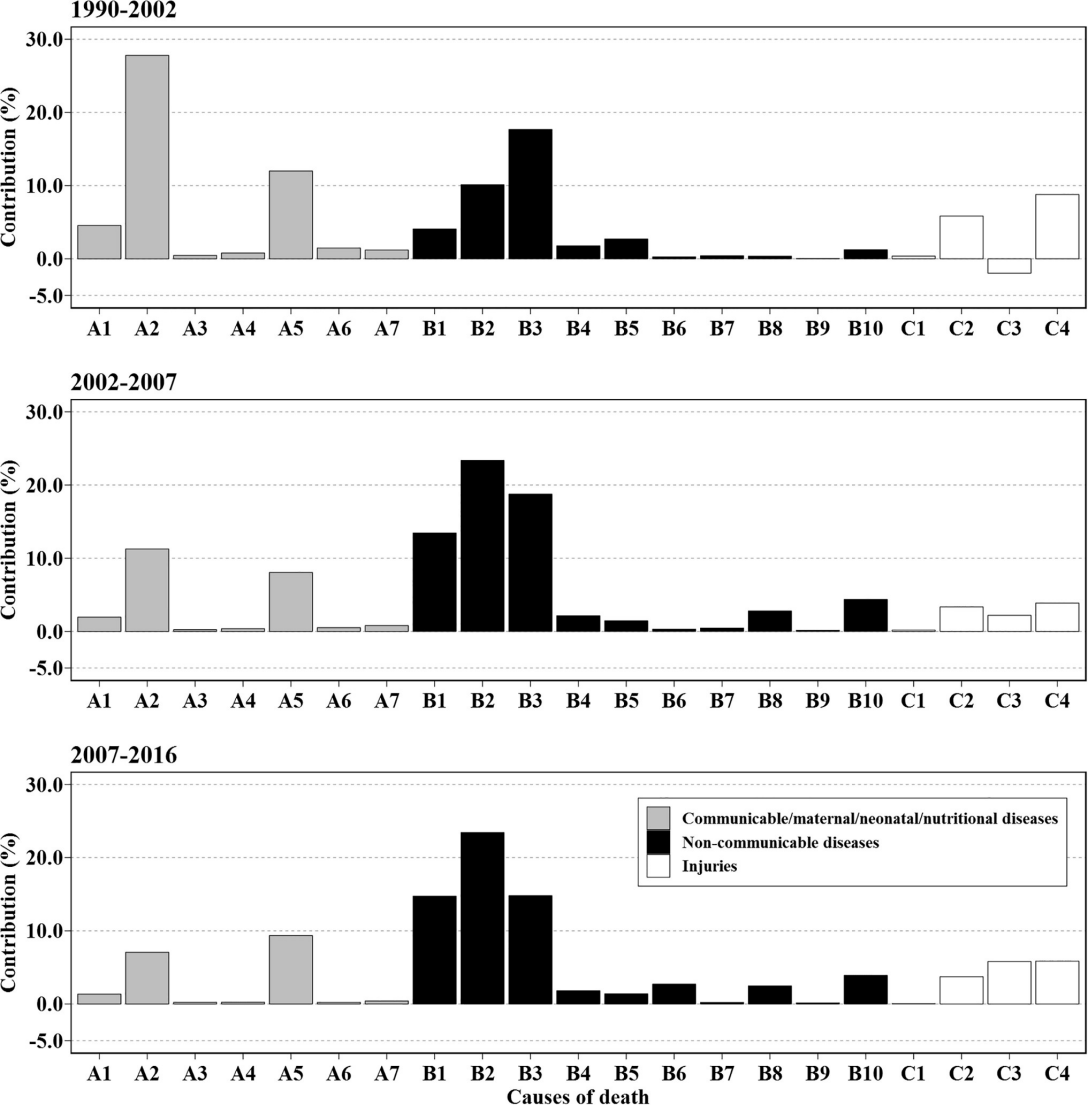
Cause-specific contributions to life expectancy in different segments. Abbreviation: A = Communicable, maternal, neonatal, and nutritional diseases (CMNNs); B = Non-communicable diseases (NCDs) and C = injuries. A1 = HIV/AIDS and tuberculosis; A2 = Diarrhea, lower respiratory, and other common infectious diseases; A3 = Neglected tropical diseases and malaria; A4 = Maternal disorders; A5 = Neonatal disorders; A6 = Nutritional deficiencies; A7 = Other communicable, maternal, neonatal, and nutritional diseases; B1 = Neoplasms; B2 = Cardiovascular diseases; B3 = Chronic respiratory diseases; B4 = Cirrhosis and other chronic liver diseases; B5 = Digestive diseases; B6 = Neurological disorders; B7 = Mental and substance use disorders; B8 = Diabetes, urogenital, blood, and endocrine diseases; B9 = Musculoskeletal disorders; B10 = Other non-communicable diseases; C1 = Forces of nature, conflict and terrorism, and executions and police conflict; C2 = Self-harm and interpersonal violence; C3 = Transport injuries; C4 = Unintentional injuries.

**Fig 4:**
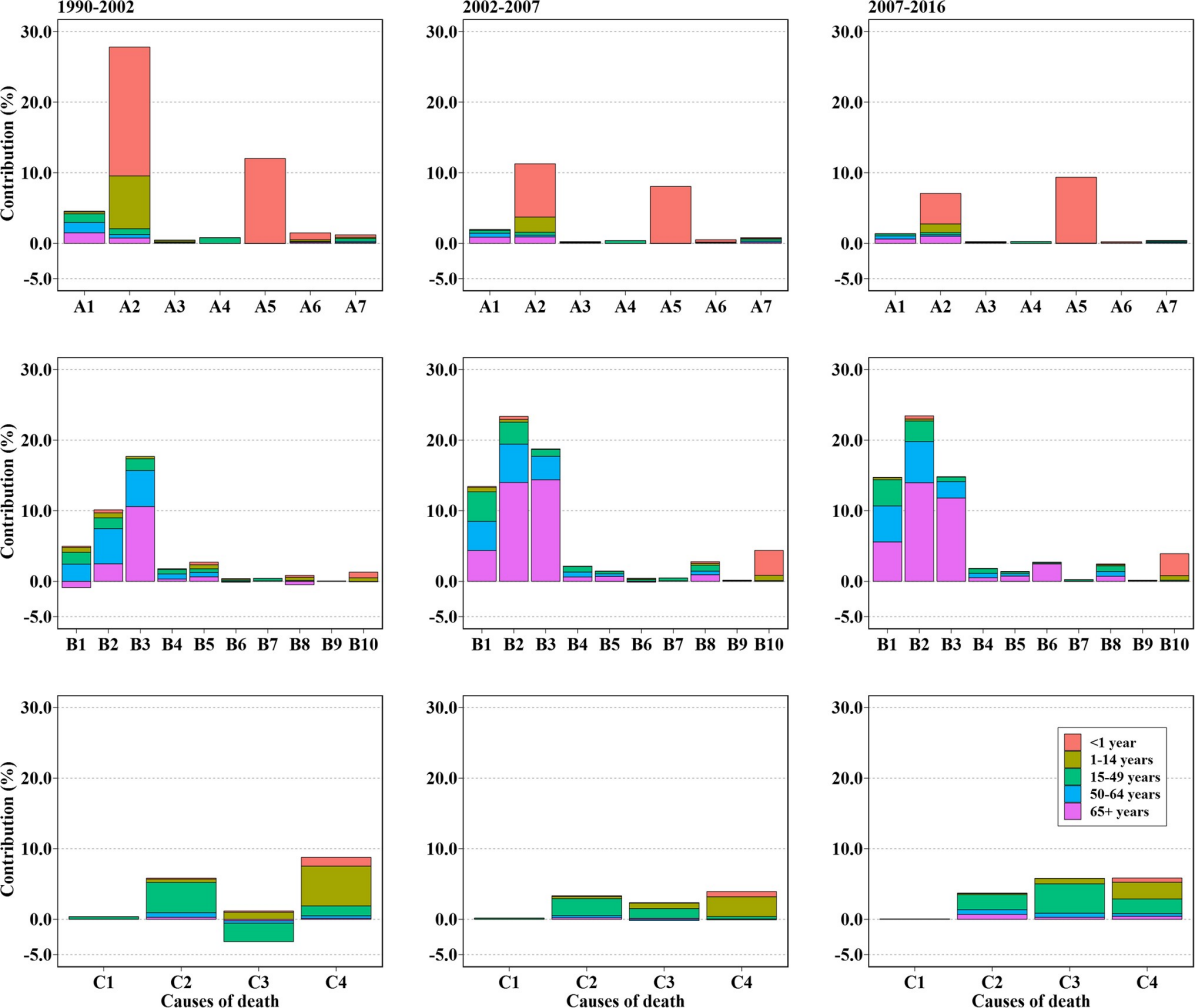
Age/Cause-specific contributions to life expectancy in different segments. Abbreviation: A = Communicable, maternal, neonatal, and nutritional diseases (CMNNs); B = Non-communicable diseases (NCDs) and C = injuries. A1 = HIV/AIDS and tuberculosis; A2 = Diarrhea, lower respiratory, and other common infectious diseases; A3 = Neglected tropical diseases and malaria; A4 = Maternal disorders; A5 = Neonatal disorders; A6 = Nutritional deficiencies; A7 = Other communicable, maternal, neonatal, and nutritional diseases; B1 = Neoplasms; B2 = Cardiovascular diseases; B3 = Chronic respiratory diseases; B4 = Cirrhosis and other chronic liver diseases; B5 = Digestive diseases; B6 = Neurological disorders; B7 = Mental and substance use disorders; B8 = Diabetes, urogenital, blood, and endocrine diseases; B9 = Musculoskeletal disorders; B10 = Other non-communicable diseases; C1 = Forces of nature, conflict and terrorism, and executions and police conflict; C2 = Self-harm and interpersonal violence; C3 = Transport injuries; C4 = Unintentional injuries.

Gains of LE attributed to different causes of death were presented in Fig 3, along with the results of different age groups (Fig 4) and sex (S2 and S3 Figs). In the first segment, increase in LE mainly resulted from diarrhea, lower respiratory, and other common infectious diseases (1.00 years, 27.79%), Chronic respiratory diseases (0.63 years, 17.68%) and neonatal disorders (0.43 years, 11.99%). As a whole, CMNNs (1.73 years, 48.27%) contributed the most for the increase to LE followed by NCDs (1.39 years, 38.69%). It's worth noting that transport injuries had negative impact on LE for those aged >15 years (-0.11 years, -3.16%), along with neoplasms (-0.03 years, -0.89%) and diabetes, urogenital, blood, and endocrine diseases (-0.02 years, -0.48%) for those older adults. For females, the contributions from cardiovascular diseases and chronic respiratory diseases were higher, which accounted for 0.59 years (13.83%) and 0.70 years (16.39%). For males, although contributions of diarrhea, lower respiratory, and other common infectious diseases were lower than that of females (0.90 vs. 1.11 years), they had a higher proportion (29.32% vs. 26.09%). Especially, the negative effect of transport injuries increased in males (-0.13 years, -4.10%).

For the rapid increase from 2002, the most prominent causes for the increases of LE were NCDs including cardiovascular diseases (0.67 years, 23.36%), chronic respiratory diseases (0.54 years, 18.76%) and neoplasms (0.39 years, 13.44%). Compared to the first segment, contributions of diabetes, urogenital, blood, and endocrine diseases and other non-communicable diseases also increased (>2.50%) as well as transport injuries which changed negative effects into positive ones. However, contributions of unintentional injuries became smaller from 8.78% to 3.87%. In general, the effect of CMNNs decreased accounting for +0.66 years (23.17%), while NCDs accounting for 1.93 years (67.24%) of the total increase in LE. Contributions from older adults (aged 65+) predominated in NCDs especially in cardiovascular diseases and chronic respiratory diseases. Moreover, similar patterns were found for males and females.

Starting in 2007, increases in LE mainly resulted from NCDs (1.95 years, 64.78%), along with the decreasing proportion of CMNNs (<20%). The rising speed in LE has slowed with declined in contributions of diarrhea, lower respiratory, and other common infectious diseases (0.22 years, 7.25%) and chronic respiratory diseases (0.46 years, 15.20%). In contrast, proportions of contributions of transport injuries and unintentional injuries increased to 5.96% and 6.02%, respectively. For females, contributions of cardiovascular diseases sharply increased LE by 1.17 years (32.26%). Among them, older adults accounted for the most (0.77 years, 21.30%). Whereas, contributions of transport injuries and unintentional injuries were larger in males. Overall, increase in LE of females was mainly explained by NCDs (2.65 years, 72.90%) which was much higher than that of males (1.43 years, 56.03%).

Detailed contributions stratified by age, cause and sex to LE gap were presented in S1 Appendix.

## Discussion

LE is a comprehensive method for overall population health for a long time. In this study, Arriaga’s decomposition method was used to investigate age and cause- specific changes of LE in China during 1990–2016. From 1990 to 2002, CMNNs played an important role in the contributions to LE which were mainly influenced by infant mortality rate. Reductions of infant mortality in diarrhea, lower respiratory, and other common infectious diseases and neonatal disorders contributed most to LE, which was consistent with results in other studies [23, 24]. Steady declines in fertility, comprehensive universal health insurance and the effective promotion of national health policy for children pediatric healthcare, including implementations of one-child family planning, immunization and nutritional support may account for the contributions from infant mortality [25–27].

After 2002, the rapid increases in LE were mainly attained by declining mortality in NCDs which came from the elderly. The impact of ageing population gradually appeared in China [28, 29]. There appeared to be a trend that the contribution to LE, centralized more from advanced age (65+ years), which was consistent in other developed countries in the early period [30–33]. The major driving force of gains in LE mainly came from constant reductions in mortality of cardiovascular diseases, chronic respiratory diseases and neoplasms, although they were still leading causes of death in China [34]. Among them, cardiovascular diseases contributed the most which was consistent with results from other countries [32, 35]. And unlike males, contributions of cardiovascular diseases for LE of females sharply increased since 2007. Reductions in mortality of cardiovascular diseases may be driven mostly by the application of a variety of treatment and prevention measures such as healthy lifestyle and Dietary change [36]. The prevalence of smoking had declined since 1996, although it is still a main risk factor of cardiovascular diseases in Chinese population [37]. In addition, chronic respiratory diseases and neoplasms also played important roles in the increased LE, mainly benefited from successful management of air pollution such as industrial upgrading, environmental health policy implementation, surveillance [38] and the strategy of early diagnosis and treatment in cancer control based on the improved surveillance network [39, 40]. However, since 2007, contributions of chronic respiratory diseases to LE for both sexes and contributions of neoplasms to LE for males decreased. So it is necessary to strengthen the strategies of prevention and control so as to have a long-term effect on chronic respiratory diseases and neoplasms.

Mortality of transport injuries increased during 1990–2002, possibly because of rapid industrialization with increased motor vehicles [41]. As a result, transport injuries had a negative impact on LE, especially for those of 15–49 years and males. After 2002, a significant and gradual decrease in the mortality of transport injuries was observed [42] which was mainly attributed to the significant legislation on road safety. In China, the Central Government has undertaken a series of strong initiatives to improve road safety, including the Road Traffic Safety Law (2004) and the Criminal Law amendment (Eight) which had strong effect on road safety [43, 44]. Since 2007, the contributions of transport injuries further increased mainly in males aged 15–49 years. More efforts are needed to integrate safety into road design, improve road traffic management, and avoid unsafe behaviors in China [45]. Moreover, due to the high proportion of drowning in unintentional injuries of those aged 1–14 years, the decreased mortality of drowning may lead to positive contributions to LE [46]. Since 1990, the Chinese government had implemented three China Children's Development Outlines (CCDOs). Each CCDO proposed corresponding main targets and strategic measures and provided important policies and impetus for the prevention and control of child injury [47]. Considering the decreasing trend of contributions of unintentional injuries after 2002, it is necessary to establish and improve systematic, comprehensive and scientific strategies for injury prevention with relevant policies and regulations based on "Healthy China 2030" to formulate and implement action plans [48].

China has experienced an epidemiological transition shifting from infectious diseases to chronic diseases in much shorter time than many other countries [4]. The pace and spread of behavioral changes, including changing diets, decreased physical activity and other high risk behaviors, has accelerated to an unprecedented degree [49]. Unfortunately, China’s ageing population structure will inevitably produce higher ratios of the chronic diseases that are commonly seen in older age groups. Declining trend is gradually weakening in NCDs such as cardiovascular diseases, chronic respiratory diseases and neoplasms which were the main barriers in the way of living longer.

There are several limitations in our study. First, the quality of mortality data varied in different segments, which might affect the accuracy of the estimated trends in LE. Second, only 3 segments were analyzed with 2 joinpoints from 1990 to 2016. Decompositions of LE gaps can provide useful information even when there appears to be little between years. In the future, age and cause-specific contributions between each year will be explored. Third, our study only included 21 causes of death. The total effects of each cause were calculated without a more detailed division. Different specific diseases may have opposite effect in the same cause. So more comprehensive studies will be in demand.

With the change of the environment and people’s lifestyle, although cardiovascular diseases, chronic respiratory diseases and neoplasms contributed mostly to the life expectancy both in males and females, they are the main causes of death yet. With the aging population, NCDs that caused by complex relationships of genetic factors, lifestyle, diet, climate and socioeconomic status are becoming dominant in the improvements of LE. These results provide rich information for setting priorities when allocating limited resources. The causes in high contributions of specific ages are of special concern in the future and targeted strategies and policies should be implemented in achieving the goal of LE in the program of “healthy China 2030”.

## Conclusions

LE in China increased rapidly during 1990–2016, and the transition from infectious diseases to non-communicable diseases already happened. More attention should be paid to NCDs such as cardiovascular diseases, chronic respiratory diseases and neoplasms which were mainly attributed to the increase of LE, especially for males and elderly population.

## Supporting information

S1 Fig Trend in life expectancy for male and female in China from 1990 to 2016. LE, life expectancy; β: slope coefficient; ^ Indicates that the slope coefficient is significantly different from zero at α = 0.05 level. Arrow indicate two joinpoints (2002 and 2007) for the trend of life expectancy from 1990 to 2016.
(TIF)

S2 Fig Cause-specific contributions to life expectancy of different age groups in different segments for females. A = Communicable, maternal, neonatal, and nutritional diseases (CMNNs); B = Non-communicable diseases (NCDs) and C = injuries. A1 = HIV/AIDS and tuberculosis; A2 = Diarrhea, lower respiratory, and other common infectious diseases; A3 = Neglected tropical diseases and malaria; A4 = Maternal disorders; A5 = Neonatal disorders; A6 = Nutritional deficiencies; A7 = Other communicable, maternal, neonatal, and nutritional diseases; B1 = Neoplasms; B2 = Cardiovascular diseases; B3 = Chronic respiratory diseases; B4 = Cirrhosis and other chronic liver diseases; B5 = Digestive diseases; B6 = Neurological disorders; B7 = Mental and substance use disorders; B8 = Diabetes, urogenital, blood, and endocrine diseases; B9 = Musculoskeletal disorders; B10 = Other non-communicable diseases; C1 = Forces of nature, conflict and terrorism, and executions and police conflict; C2 = Self-harm and interpersonal violence; C3 = Transport injuries; C4 = Unintentional injuries.
(TIFF)

S3 Fig Cause-specific contributions to life expectancy of different age groups in different segments for males. A = Communicable, maternal, neonatal, and nutritional diseases (CMNNs); B = Non-communicable diseases (NCDs) and C = injuries. A1 = HIV/AIDS and tuberculosis; A2 = Diarrhea, lower respiratory, and other common infectious diseases; A3 = Neglected tropical diseases and malaria; A4 = Maternal disorders; A5 = Neonatal disorders; A6 = Nutritional deficiencies; A7 = Other communicable, maternal, neonatal, and nutritional diseases; B1 = Neoplasms; B2 = Cardiovascular diseases; B3 = Chronic respiratory diseases; B4 = Cirrhosis and other chronic liver diseases; B5 = Digestive diseases; B6 = Neurological disorders; B7 = Mental and substance use disorders; B8 = Diabetes, urogenital, blood, and endocrine diseases; B9 = Musculoskeletal disorders; B10 = Other non-communicable diseases; C1 = Forces of nature, conflict and terrorism, and executions and police conflict; C2 = Self-harm and interpersonal violence; C3 = Transport injuries; C4 = Unintentional injuries.
(TIF)

S1 Table List of International Classification of Diseases (ICD) codes for causes of death from Global Burden of Disease 2016. A, communicable, maternal, neonatal, and nutritional diseases; B, non-communicable diseases; C, injuries.
(DOCX)

S2 Table Estimated regression coefficients for different phases. 2 joinpoints were identified in the trend of life expectancy in China. Three segments were 1990–2002, 2002–2007 and 2007–2016. Linear model was applied for each segment.
(DOCX)

S1 Appendix **Section 1: Cause of death data standardisation, processing and estimation**. Section 2: Detailed contributions stratified by age, cause and sex to life expectancy gap.
(DOCX)

S1 Data (XLSX)
