# Supplementary figures and images for: Patterns and changes in life expectancy in China, 1990-2016
Source: PLoS One. 2020 Apr 1;15(4):e0231007. doi: 10.1371/journal.pone.0231007 (PMC7112202; doi:10.1371/journal.pone.0231007)

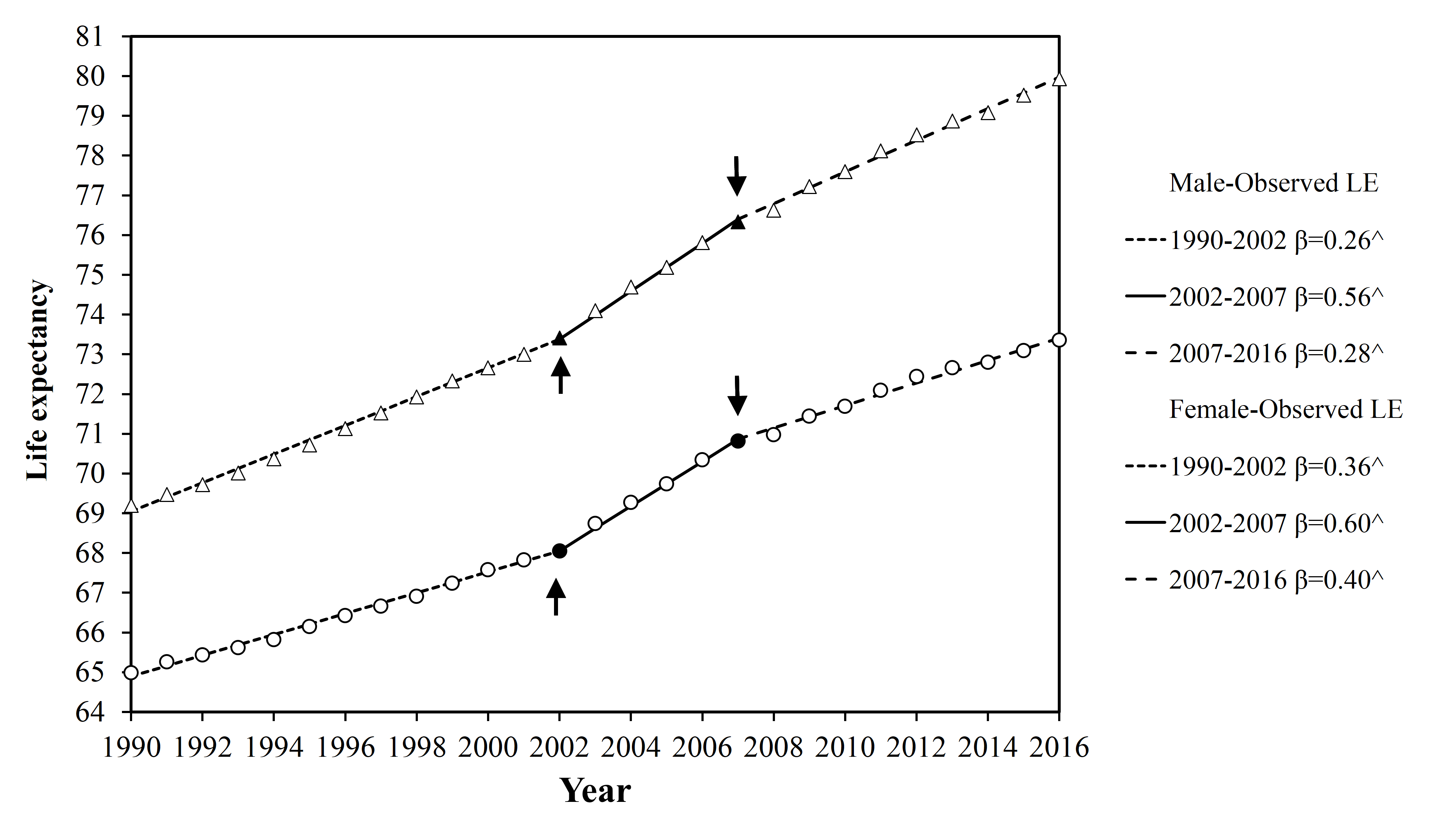

Supplement: S1 Fig — LE, life expectancy; β: slope coefficient; ^ Indicates that the slope coefficient is significantly different from zero at α = 0.05 level. Arrow indicate two joinpoints (2002 and 2007) for the trend of life expectancy from 1990 to 2016. (TIF) [file pone.0231007.s001.tif]

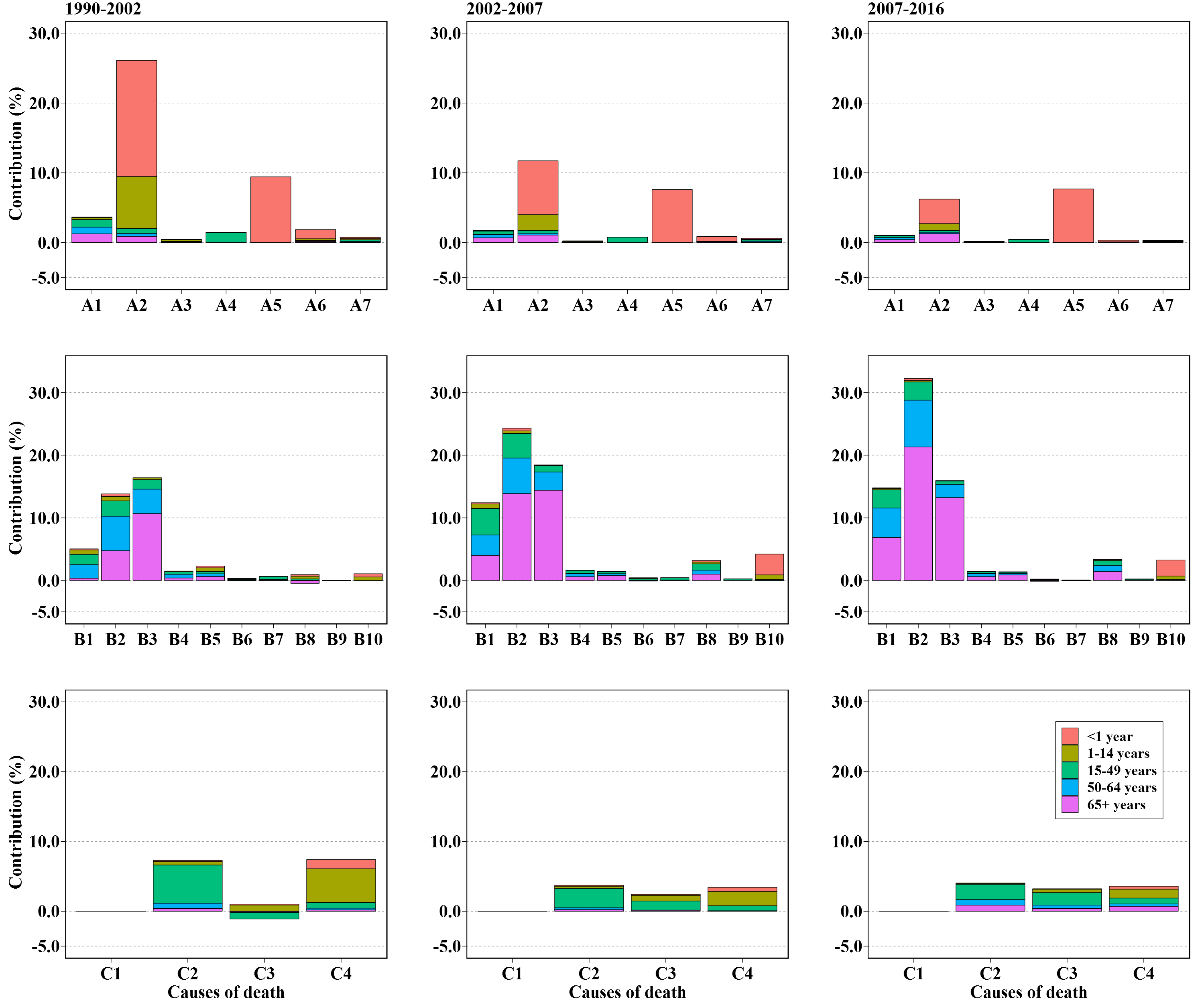

Supplement: S2 Fig — A = Communicable, maternal, neonatal, and nutritional diseases (CMNNs); B = Non-communicable diseases (NCDs) and C = injuries. A1 = HIV/AIDS and tuberculosis; A2 = Diarrhea, lower respiratory, and other common infectious diseases; A3 = Neglected tropical diseases and malaria; A4 = Maternal disorders; A5 = Neonatal disorders; A6 = Nutritional deficiencies; A7 = Other communicable, maternal, neonatal, and nutritional diseases; B1 = Neoplasms; B2 = Cardiovascular diseases; B3 = Chronic respiratory diseases; B4 = Cirrhosis and other chronic liver diseases; B5 = Digestive diseases; B6 = Neurological disorders; B7 = Mental and substance use disorders; B8 = Diabetes, urogenital, blood, and endocrine diseases; B9 = Musculoskeletal disorders; B10 = Other non-communicable diseases; C1 = Forces of nature, conflict and terrorism, and executions and police conflict; C2 = Self-harm and interpersonal violence; C3 = Transport injuries; C4 = Unintentional injuries. (TIFF) [file pone.0231007.s002.tiff]

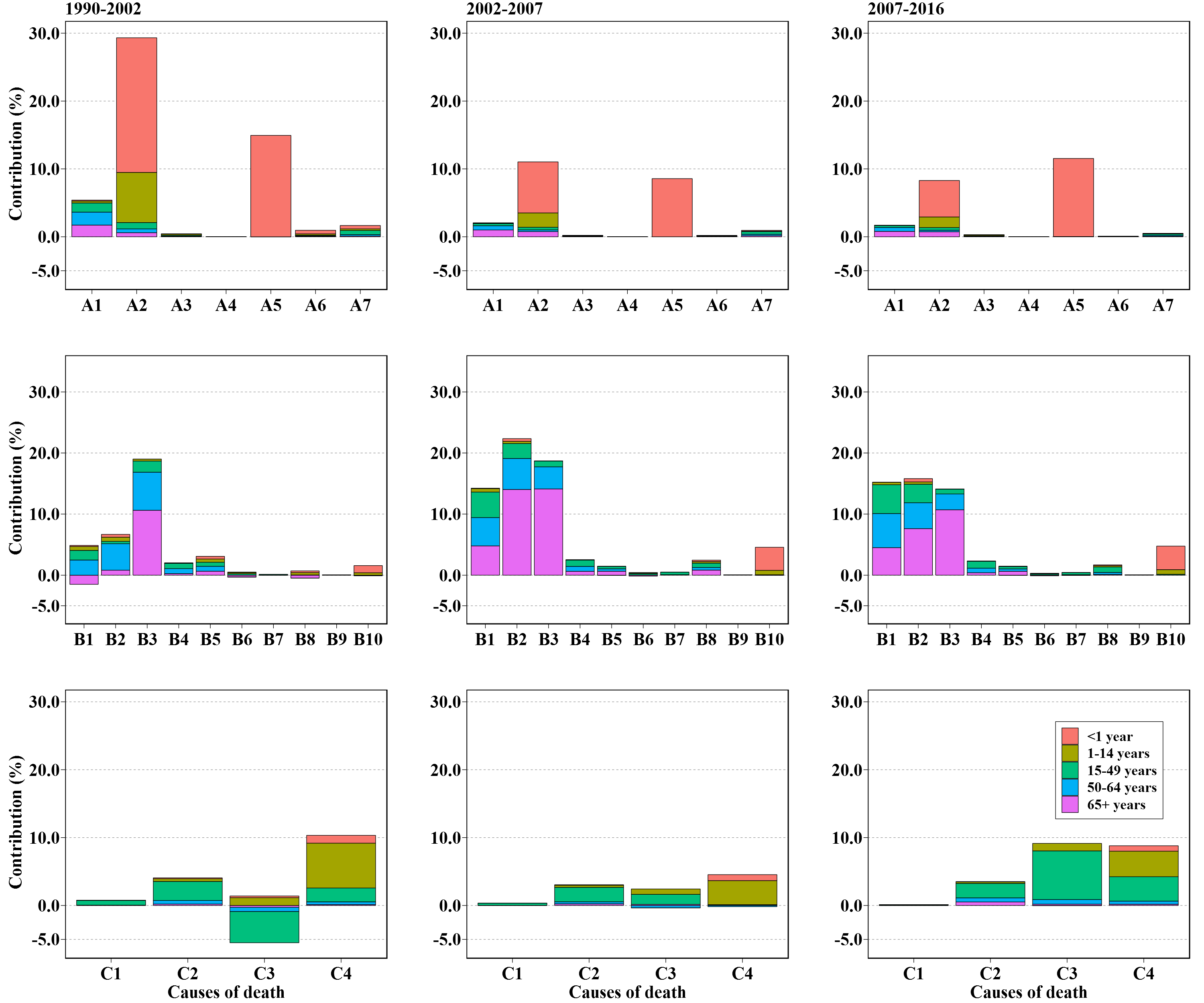

Supplement: S3 Fig — A = Communicable, maternal, neonatal, and nutritional diseases (CMNNs); B = Non-communicable diseases (NCDs) and C = injuries. A1 = HIV/AIDS and tuberculosis; A2 = Diarrhea, lower respiratory, and other common infectious diseases; A3 = Neglected tropical diseases and malaria; A4 = Maternal disorders; A5 = Neonatal disorders; A6 = Nutritional deficiencies; A7 = Other communicable, maternal, neonatal, and nutritional diseases; B1 = Neoplasms; B2 = Cardiovascular diseases; B3 = Chronic respiratory diseases; B4 = Cirrhosis and other chronic liver diseases; B5 = Digestive diseases; B6 = Neurological disorders; B7 = Mental and substance use disorders; B8 = Diabetes, urogenital, blood, and endocrine diseases; B9 = Musculoskeletal disorders; B10 = Other non-communicable diseases; C1 = Forces of nature, conflict and terrorism, and executions and police conflict; C2 = Self-harm and interpersonal violence; C3 = Transport injuries; C4 = Unintentional injuries. (TIF) [file pone.0231007.s003.tif]
