# Supplementary material for: Patterns and changes in life expectancy in China, 1990-2016
Source: PLoS One. 2020 Apr 1;15(4):e0231007. doi: 10.1371/journal.pone.0231007 (PMC7112202; doi:10.1371/journal.pone.0231007)
Supplement: S1 Table — A, communicable, maternal, neonatal, and nutritional diseases; B, non-communicable diseases; C, injuries. (DOCX) [file pone.0231007.s004.docx]

**S1 Table | List of International Classification of Diseases (ICD) codes for causes of death from Global Burden of Disease 2016.**

| **Causes of death** | | **ICD10** | **ICD9** |
| --- | --- | --- | --- |
| A1 | HIV/AIDS and tuberculosis | A10-A14, A15-A19.9, B20-B24.9, B90-B90.9, K67.3, K93.0, M49.0, N74.1-N74.2, P37.0, U84.3 | 010-019.9, 042-044.9, 137-137.9, 138.0-138.9, 730.4-730.6 |
| A2 | Diarrhea, lower respiratory, and other common infectious diseases | A00-A00.9, A01.0-A09.9, A33-A37.9, A39-A39.9, A48.1, A70, A83-A87.9, B01-B02.9, B05-B05.9, B94.1, B97.4-B97.6, F07.1, G00.0-G00.8, G03-G03.8, G04-G05.8, H70-H70.9, J01-J01.9, J04.0, J05-J05.0, J09-J15.8, J16-J16.9, J20-J21.9, J36-J36.0, P23.0-P23.4, P35.8, R19.7, U04-U04.9 | 001-001.9, 002.0-009.9, 032-033.9, 036-036.3, 036.5-037.9, 047-049.9, 052-053.9, 055-055.9, 062-064.9, 079.6, 139.0, 320.0-320.8, 321-323, 323.4-323.9, 381-383.9, 461-461.9, 464.0, 464.2, 464.4, 464.8-464.9, 466-469, 470.0, 475-475.9, 476.9, 480-482.8, 483.0-483.9, 484.0-484.3, 484.6-484.7, 487-489, 771.3 |
| A3 | Neglected tropical diseases and malaria | A30-A30.9, A68-A68.9, A69.2-A69.9, A75-A75.9, A77-A79.9, A82-A82.9, A90-A96.9, A98-A98.8, B33.0-B33.1, B50-B53.8, B55.0, B56-B57.5, B60-B60.8, B65-B67.9, B69-B72.0, B74.3-B75, B77-B77.9, B83-B83.8, B92, K93.1, P37.1, U06-U06.9 | 030-030.9, 060-061.8, 065-066.9, 071-071.9, 080-083.9, 084.0-084.5, 084.7-084.9, 085.0, 086-088, 088.8-088.9, 120-124.9, 125.4-125.9, 127-127.1, 128-129.0, 425.6 |
| A4 | Maternal disorders | N96, N98-N98.9, O00-O07.9, O09-O16.9, O20-O26.9, O28-O36.9, O40-O48.1, O60-O77.9, O80-O92.7, O96-O98.6, O98.8-O99.9 | 630-636.9, 638-638.9, 640-679.1 |
| A5 | Neonatal disorders | P00-P04.2, P04.5-P05.9, P07-P15.9, P19-P22.9, P24-P29.9, P36-P36.9, P38-P39.9, P50-P61.9, P70, P70.3-P72.9, P74-P78.9, P80-P81.9, P83-P84, P90-P94.9, P96, P96.3-P96.4, P96.8 | 760-760.6, 760.8-768, 768.2-770, 770.1-771, 771.4-775, 775.4-779.3, 779.6-779.8 |
| A6 | Nutritional deficiencies | D50.1-D50.8, D51-D52.0, D52.8-D53.9, D64.3, E00-E02, E40-E46.9, E51-E61.9, E63-E64.0, E64.2-E64.9, M12.1 | 244.2, 260-263.9, 265-269.9, 280.1-280.8, 281.0-281.9, 716.0 |
| A7 | Other communicable, maternal, neonatal, and nutritional diseases | A20-A28.9, A32-A32.9, A38-A38.9, A48.2, A48.4-A48.5, A49.1, A50-A58, A60-A60.9, A63-A63.8, A65-A65.0, A69-A69.1, A74, A74.8-A74.9, A80-A81.9, A88-A89.9, B00-B00.9, B03-B04, B06-B06.9, B10-B10.8, B15-B17.9, B19-B19.9, B25-B27.9, B29.4, B33, B33.3-B33.8, B47-B48.8, B63, B91, B94.2, B95-B95.5, G14-G14.6, I00, I02, I02.9, I98.0-I98.1, J02.0, J03.0, K67.0-K67.2, K67.8, K71.2, K71.6, K74.7-K74.8, K75.3, K76.3, K77.0, M03.1, M49.1, M73.0-M73.1, M89.6, P35-P35.3, P35.9, P37, P37.2, P37.5-P37.9, U82-U84, U85-U89, Z16-Z16.3 | 020-029, 034-034.9, 040, 040.1-041.0, 045-046.9, 050-051.9, 054-054.9, 056-059.9, 070-070.9, 072-074.1, 074.3-075.9, 078.3-078.7, 079-079.5, 079.7, 090-101.6, 104-104.9, 136-136.2, 138, 139, 323.0-323.3, 390-390.9, 392, 392.9, 484.4-484.5, 771.0-771.2, V09-V09.9 |
| B1 | Neoplasms | C00-C13.9, C15-C25.9, C30-C34.9, C37-C38.8, C40-C41.9, C43-C45.9, C47-C54.9, C56-C57.8, C58-C58.0, C60-C63.8, C64-C67.9, C68.0-C68.8, C69-C75.8, C81-C86.6, C88-C96.9, D00.1-D00.2, D01.0-D01.3, D02.0-D02.3, D03-D06.9, D07.0-D07.2, D07.4-D07.5, D09.0, D09.2-D09.3, D09.8, D10.0-D10.7, D11-D12.9, D13.0-D13.7, D14.0-D14.3, D15-D16.9, D22-D24.9, D26.0, D27-D27.9, D28.0-D28.1, D28.7, D29.0-D29.8, D30.0-D30.8, D31-D36, D36.1-D36.7, D37.1-D37.5, D38.0-D38.5, D39.1-D39.2, D39.8, D40.0-D40.8, D41.0-D41.8, D42-D43.9, D44.0-D44.8, D45-D47.0, D47.2-D47.9, D48.0-D48.6, D49.2-D49.4, D49.6, K31.7, K62.0-K62.1, K63.5, N60-N60.9, N84.0-N84.1, N87-N87.9 | 140-148.9, 150-158.9, 160-164.9, 170-175.9, 180-183.8, 184.0-184.4, 184.8, 185-186.9, 187.1-187.8, 188-188.9, 189.0-189.8, 190-194.8, 200-208.9, 209.0-209.1, 209.4-209.5, 210.0-210.9, 211.0-211.8, 212.0-212.8, 213-213.9, 217-217.8, 219.0, 220-220.9, 221.0-221.8, 222.0-222.8, 223.0-223.8, 224-228.9, 229.0, 229.8, 230.1-230.8, 231.0-231.2, 232-232.9, 233.0-233.2, 233.4-233.5, 233.7, 234.0-234.8, 235.0, 235.4, 235.6-235.8, 236.1-236.2, 236.4-236.5, 236.7, 237-237.3, 237.5-237.9, 238.0-238.9, 239.2-239.4, 239.6, 569.0, 610-610.9 |
| B2 | Cardiovascular diseases | B33.2, G45-G46.8, I01-I01.9, I02.0, I05-I09.9, I11-I11.9, I20-I25.9, I28-I28.8, I30-I31.1, I31.8-I41.9, I42.1-I42.8, I43-I43.9, I47-I48.9, I51.0-I51.4, I60-I63.9, I65-I66.9, I67.0-I67.3, I67.5-I67.6, I68.0-I68.2, I69.0-I69.3, I70.2-I70.8, I71-I73.9, I77-I83.9, I86-I89.0, I89.9, I98, K75.1 | 036.4, 074.2, 391-391.9, 392.0, 393-398.9, 402-402.9, 410-414.9, 417-417.9, 420-423, 423.1-424.9, 425.0-425.3, 425.5, 425.7-425.8, 427-427.3, 427.6-427.8, 429.0, 430-435.9, 437.0-437.2, 437.5-437.8, 440.2, 440.4, 441-443.9, 447-454.9, 456, 456.3-457, 457.1, 457.8-457.9, 459, 459.1-459.3 |
| B3 | Chronic respiratory diseases | D86-D86.2, D86.9, G47.3, J30-J35.9, J37-J47.9, J60-J63.8, J65-J68.9, J70-J70.1, J70.8-J70.9, J82, J84-J84.9, J91-J92.9 | 135-135.9, 136.6, 327.2-327.8, 470, 470.9-474.9, 476-476.1, 477-479, 490-504.9, 506-506.9, 508-509, 515, 516-517.8, 518.6, 518.9, 519.1-519.4 |
| B4 | Cirrhosis and other chronic liver diseases | B18-B18.9, I85-I85.9, I98.2, K70-K70.9, K71.3-K71.5, K71.7, K72.1-K74.6, K74.9, K75.2, K75.4-K76.2, K76.4-K76.9, K77.8 | 456.0-456.2, 571-571.9, 572.2-573.9 |
| B5 | Digestive diseases | I84-I84.9, K20-K29.9, K31-K31.6, K31.8, K35-K38.9, K40-K42.9, K44-K46.9, K50-K52.9, K55-K62, K62.2-K62.6, K62.8-K62.9, K64-K64.9, K66.8, K67, K68-K68.9, K77, K80-K83.9, K85-K86.9, K90-K90.9, K92.8, K93.8, M09.1 | 455-455.9, 530-536.1, 537-537.6, 537.8, 538, 540-543.9, 550-551.1, 551.3-552.1, 552.3-553.6, 555-558.9, 560-560.3, 560.8-560.9, 562-562.1, 564-564.1, 564.5-564.7, 565-566.9, 569.1-569.5, 569.7, 574-577.9, 579-579.2, 579.4-579.9, 787.1 |
| B6 | Neurological disorders | F00-F03.9, G10-G13.8, G20-G21.0, G21.2-G24, G24.1-G25.0, G25.2-G25.3, G25.5, G25.8-G26.0, G30-G31.1, G31.8-G31.9, G35-G37.9, G40-G41.9, G61-G61.9, G70-G72, G72.2-G73.7, G90-G90.9, G95-G95.9, M33-M33.9 | 290-290.9, 294.1-294.9, 330-331.2, 331.5-337.9, 340-341.9, 345-345.9, 349, 349.2-349.8, 353.6-353.9, 356-356.9, 357.0-357.1, 357.3-357.4, 357.7, 358-359.9, 775.2 |
| B7 | Mental and substance use disorders | F10-F16.9, F18-F19.9, F24, F50.0-F50.5, G31.2, G72.1, P04.3-P04.4, P96.1, Q86.0, R78.0-R78.5, X45-X45.9, X65-X65.9, Y15-Y15.9 | 291-292.9, 303-303.9, 304.0-304.8, 305-305.9, 307.1, 357.5, 760.7, 790.3, E850-E854, E860 |
| B8 | Diabetes, urogenital, blood, and endocrine diseases | D25-D26, D26.1-D26.9, D28.2, D52.1, D55-D58.9, D59.0-D59.3, D59.5-D59.6, D60-D61.9, D63.1, D64.0, D64.4, D66-D67, D68.0-D69.8, D70-D75.8, D76-D78.8, D86.8, D89-D89.3, E03-E07.1, E09-E14.9, E15.0, E16.0-E16.9, E20-E34.8, E36-E36.8, E65-E68, E70-E85.2, E88-E89.9, G21.1, G24.0, G25.1, G25.4, G25.6-G25.7, G72.0, G93.7, G97-G97.9, I12-I13.9, I95.2-I95.3, I97-I97.9, I98.9, J70.2-J70.5, J95-J95.9, K43-K43.9, K62.7, K91-K91.9, K94-K95.8, M87.1, N00-N08.8, N10-N12.9, N14-N16.8, N18-N18.9, N20-N23.0, N25-N28.1, N29-N32.0, N32.3-N32.4, N34-N34.3, N36-N36.9, N39-N39.2, N41-N41.9, N44-N44.0, N45-N45.9, N49-N49.9, N65-N65.1, N72-N72.0, N75-N77.8, N80-N81.9, N83-N83.9, N99-N99.9, P70.0-P70.2, P96.2, P96.5, Q61-Q62.8, R50.2, R73-R73.9 | 218-219, 219.1-219.9, 236.0, 240-243.9, 244.0-244.1, 244.3-244.8, 245-246.9, 250-259.9, 270-273.9, 275-276, 277-277.2, 277.4-277.9, 278.0-278.8, 282-284.9, 286-286.5, 286.7-289.7, 357.2, 357.6, 403-404.9, 518.7, 519.0, 536.4, 539-539.9, 551.2, 552.2, 564.2-564.4, 569.6, 579.3, 580-583.9, 585-585.9, 588-590.9, 592-593.8, 594-599.6, 599.8, 601-602.9, 604-604.9, 608.2, 617-618.9, 620-620.9, 621.4-621.9, 622.3-622.7, 629-629.8, 753-753.3, 775.0-775.1, 775.3, 779.4-779.5, 788.0, 790.2 |
| B9 | Musculoskeletal disorders | I27.1, I67.7, L93-L93.2, M00-M03.0, M03.2-M03.6, M05-M09.0, M09.2-M09.8, M30-M32.9, M34-M36.8, M40-M43.1, M65-M65.0, M71.0-M71.1, M80-M82.8, M86.3-M86.4, M87-M87.0, M88-M89.0, M89.5, M89.7-M89.9 | 416.1, 437.4, 446-446.9, 695.4-695.5, 710-711.9, 714-714.3, 714.8-714.9, 730.1, 732-732.9, 733.0-733.1 |
| B10 | Other non-communicable diseases | A46-A46.0, A66-A67.9, B86, D86.3, H05.0-H05.1, I89.1-I89.8, L00-L05.9, L08-L08.9, L10-L14.0, L51-L51.9, L88-L89.9, L97-L98.4, M72.5-M72.6, P96.0, Q00-Q07.9, Q10.4-Q18.9, Q20-Q28.9, Q30-Q36, Q37-Q45.9, Q50-Q60.6, Q63-Q86, Q86.1-Q87.8, Q89-Q89.8, Q90-Q93.9, Q95-Q99.8, R95-R95.9 | 035-035.9, 102-103.9, 133-133.6, 376.0-376.1, 457.2-457.3, 680-689, 694-695.3, 707-707.9, 740-749.0, 749.2-752.9, 753.4-758.9, 759.0-759.8, 798-798.0 |
| C1 | Forces of nature, conflict and terrorism, and executions and police conflict | U00-U03, X33-X38.9, Y35-Y38.9, Y89.0-Y89.1 | E907-E909, E970-E979, E990-E999 |
| C2 | Self-harm and interpersonal violence | X60-X64.9, X66-Y08.9, Y87.0-Y87.1 | E950-E969 |
| C3 | Transport injuries | V00-V86.9, V87.2-V87.3, V88.2-V88.3, V90-V98.8 | E800-E807, E830-E838, E840-E849 |
| C4 | Unintentional injuries | L55-L55.9, L56.3, L56.8-L56.9, L58-L58.9, W00-W46.2, W49-W62.9, W64-W70.9, W73-W75.9, W77-W81.9, W83-W94.9, W97.9, W99-X06.9, X08-X32.9, X39-X39.9, X46-X47, X47.1-X47.8, X48-X48.9, X50-X54.9, X57-X58.9, Y40-Y84.9, Y88-Y88.3 | 349.0-349.1, 457.0, E856-E857, E861-E869, E870-E876, E878-E879, E880-E886, E888-E906, E910-E928, E930-E949 |

*Note: A, communicable, maternal, neonatal, and nutritional diseases; B, non-communicable diseases; C, injuries.*
