# Supplementary material for: Patterns and changes in life expectancy in China, 1990-2016
Source: PLoS One. 2020 Apr 1;15(4):e0231007. doi: 10.1371/journal.pone.0231007 (PMC7112202; doi:10.1371/journal.pone.0231007)
Supplement: S2 Table — 2 joinpoints were identified in the trend of life expectancy in China. Three segments were 1990–2002, 2002–2007 and 2007–2016. Linear model was applied for each segment. (DOCX) [file pone.0231007.s005.docx]

**S2 Table | Estimated regression coefficients for different phases.**

| **Groups** | ***Parameter Estimate*** | | **Standard Error** | **95% CI** | **Test Statistic (t)** | **Prob > \|t\|** |
| --- | --- | --- | --- | --- | --- | --- |
| Both | Intercept 1 | -541.54 | 18.88 | [-578.54, -504.55] | -28.69 | <0.01 |
|  | Intercept 2 | -1099.06 | 68.66 | [-1233.63, -964.49] | -16.01 | <0.01 |
|  | Intercept 3 | -589.41 | 27.45 | [-643.2, -535.61] | -21.47 | <0.01 |
|  | Slope 1 | 0.31* | 0.01 | [0.29, 0.32] | 32.33 | <0.01 |
|  | Slope 2 | 0.58* | 0.03 | [0.52, 0.65] | 17.06 | <0.01 |
|  | Slope 3 | 0.33* | 0.01 | [0.30, 0.36] | 24.21 | <0.01 |
| Female | Intercept 1 | -650.00 | 19.23 | [-687.68, -612.32] | -33.81 | <0.01 |
|  | Intercept 2 | -1134.45 | 73.75 | [-1279.01, -989.89] | -15.38 | <0.01 |
|  | Intercept 3 | -720.23 | 28.23 | [-775.56, -664.89] | -25.51 | <0.01 |
|  | Slope 1 | 0.36* | 0.01 | [0.34, 0.38] | 37.51 | <0.01 |
|  | Slope 2 | 0.60* | 0.04 | [0.53, 0.68] | 16.40 | <0.01 |
|  | Slope 3 | 0.40* | 0.01 | [0.37, 0.42] | 28.28 | <0.01 |
| Male | Intercept 1 | -458.09 | 18.59 | [-494.53, -421.64] | -24.64 | <0.01 |
|  | Intercept 2 | -1058.40 | 67.87 | [-1191.42, -925.39] | -15.60 | <0.01 |
|  | Intercept 3 | -496.56 | 27.06 | [-549.59, -443.53] | -18.35 | <0.01 |
|  | Slope 1 | 0.26* | 0.01 | [0.24, 0.28] | 28.21 | <0.01 |
|  | Slope 2 | 0.56* | 0.03 | [0.50, 0.63] | 16.62 | <0.01 |
|  | Slope 3 | 0.28* | 0.01 | [0.26, 0.31] | 21.02 | <0.01 |

*Note: 2 joinpoints were identified in the trend of life expectancy in China. Three segments were 1990-2002, 2002-2007 and 2007-2016. Linear model was applied for each segment.*
