# Supplementary material for: Patterns and changes in life expectancy in China, 1990-2016
Source: PLoS One. 2020 Apr 1;15(4):e0231007. doi: 10.1371/journal.pone.0231007 (PMC7112202; doi:10.1371/journal.pone.0231007)
Supplement: S1 Appendix — Section 1: Cause of death data standardisation, processing and estimation. Section 2: Detailed contributions stratified by age, cause and sex to life expectancy gap. (DOCX) [file pone.0231007.s006.docx]

**Supplementary Information**

**Section 1: Cause of death data standardisation, processing and estimation**

**
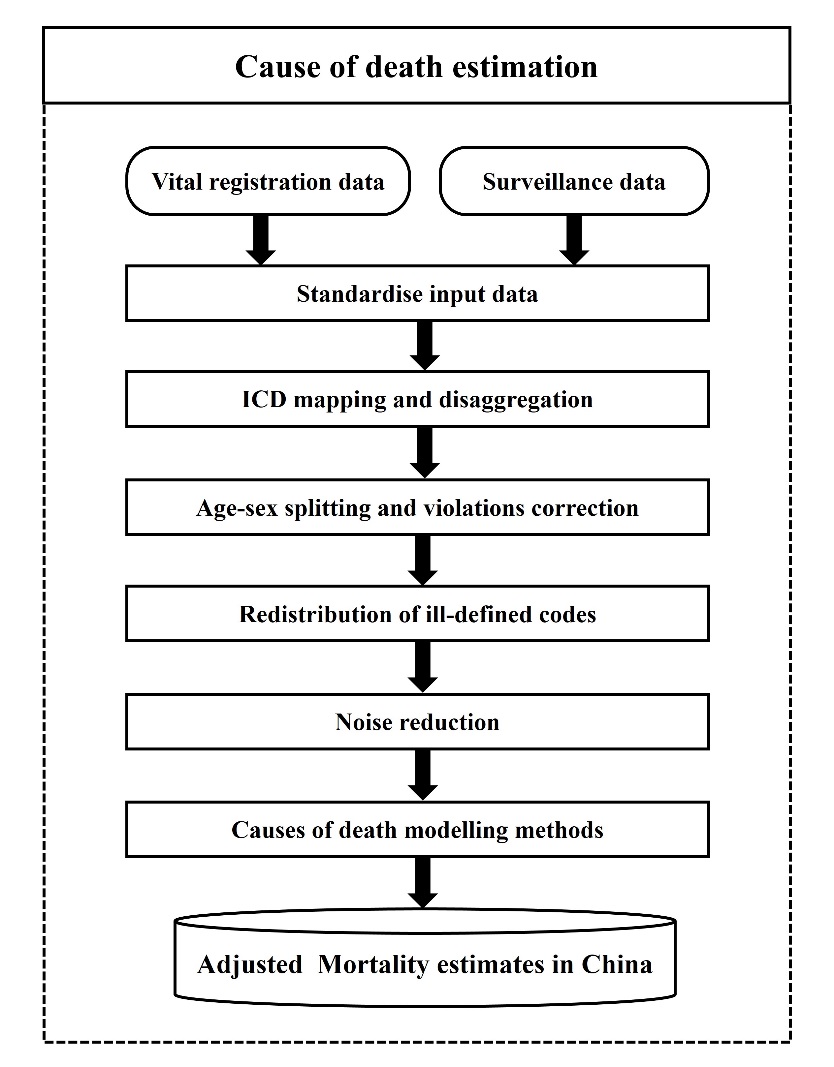
**

**Step 1. Standardise input data**

The methods of cause-of-death assignment and data collection are reviewed to determine which source type to assign; for example, we distinguish sibling history data from surveys with a verbal autopsy module. Only data at the most detailed level of the Global Burden of Disease location hierarchy are used. Documentation from the source is reviewed to determine if the population is representative of the location or only a subset of the population in that location which is used by CODEm to increase the variance associated with such data points.

Diagnostics are reviewed at this stage to avoid sending cleaning errors downstream. We review cause-specific deaths for each demographic group to ensure the data are reasonable. For example, it is unlikely that male breast cancer deaths are higher than female breast cancer or deaths from neonatal causes occur in age groups over one year. All deaths totals are compared with the sum of cause-specific deaths to ensure the observed deaths are accounted for and sample size is complete.

**Step 2. Cause of death mapping and disaggregation**

The two primary sources of data for China are surveillance data from the China Disease Surveillance Points (DSP) system and vital registration data collected by the Chinese Center for Disease Control and Prevention (CDC). In the China DSP data, deaths were reported across 145 disease surveillance points used from 1991 to 2003, 161 points used from 2004 to 2007, and 605 disease surveillance points from 2008 to 2015. While China DSP with ICD10 coding is considered sample vital registration data, it provides national coverage and cause detail. Thus it receives similar processing and treatment to the China CDC vital registration from 2008 to 2015. From 2008 to 2015, all of the deaths and cause of death information from the Disease Surveillance Points system and other system points throughout China were collected and reported via the Mortality Registration and Reporting System, an online reporting system of the Chinese CDC.

**The data used to estimate causes of death in China for GBD 2016 fell into 3 separate ICD coding schemes.**

1. The first data source, China Center for Disease Control and Prevention (CDC) Cause of Death Reporting System (2008‐2012) had ICD10 detail codes, which we mapped to the GBD cause list directly, based on our ICD 10 map.
2. The second source, Disease Surveillance Points (after 2004) had ICD 10 aggregated codes only. This meant each DSP code was defined based on an aggregated ICD10 code, aggregated from a combination of ICD 10 detailed codes. Due to the fact that we had detailed cause codes from China CDC Cause of Death Reporting System data, we were able to disaggregate the aggregated ICD 10 codes in the DSP data based on the fraction attributed to detailed cause codes from the China CDC Cause of Death Reporting System. This disaggregation only occurred for some DSP codes, which were not easily mapped to a GBD cause, such as C00-C14 which we disaggregated and mapped to GBD causes, mouth cancer, as well as Nasopharynx and other pharynx cancer.
3. The third source was also DSP, (1991-2002) which had ICD 9 aggregated codes. With this source we had a list of a combination of ICD9 codes for each DSP cause. In every DSP code that matched our standard mapping for ICD 9 codes to GBD causes we used our standard mapping. For the DSP codes that did not match a GBD cause we made bridge maps between ICD9 and ICD10 and then disaggregated the DSP codes by the ICD10 detailed pattern from the China CDC Cause of death Reporting System.

**Step 3. Age-sex splitting and violations correction**

Different sources, particularly verbal autopsy studies, report deaths for a wide range of age groups with varying intervals. For the analysis of causes of death, we mapped these different age intervals to the GBD standard set of age groups.

Occasionally, data sources will include deaths by a cause for which there is medical consensus that death is impossible for the sex and age. For example, there may be some number of deaths due to cervical cancer in males, or deaths due to maternal causes in ages under 10. When deaths violate these restrictions, we redistribute them proportionally onto all causes.

**Step 4. Redistribution of ill-defined codes**

A crucial aspect of enhancing the comparability of data for cause of death is to deal with uninformative, so-called garbage codes. Garbage codes are codes to which deaths were assigned that cannot or should not be considered as the underlying cause of death, for example: heart failure, ill-defined cancer site, senility, ill‐defined external causes of injuries, and septicaemia. The methods for redistributing these garbage-coded deaths were outlined in detail in ***Naghavi et al***.

**Four categories of garbage codes** were identified:

1. Causes that cannot or should not be considered as underlying causes of death. These are codes that are included in the ICD because of its use for classifying health service encounters but that do not signify underlying cause of death. Examples of this type of GC are all the codes under chapter 18 of ICD-10 or R codes. This category also includes two special cases in the cardiovascular area: essential primary hypertension and atherosclerosis. Essential primary hypertension is included in the ICD to classify clinical encounters, but for most physicians, it should be considered a risk factor for cardiovascular disease and not the underlying cause. This distinction between what is a risk factor and what is an underlying cause is somewhat arbitrary but necessary to enhance comparability across revisions. Finally, we included in this category a number of causes that are described as the long-term sequelae of disease, such as G82, paraplegia and tetraplegia, or O94, sequelae of complication of pregnancy, childbirth, and the puerperium.
2. Intermediate causes of death such as heart failure, septicemia, peritonitis, osteomyelitis, or pulmonary embolism. These are clearly defined clinical entities, but each has an underlying cause that would have precipitated the chain of events leading to death. Physicians who have not been adequately trained in the principles of the ICD underlying cause of death often use these causes on death certificates.
3. Immediate causes of death that are the final steps in a disease pathway leading to death. Examples of this include disseminated intravascular coagulation or defibrination syndrome (D65). The pathway to death includes the final immediate cause, an intermediate cause, and the underlying cause that triggered the chain of events. Cardiac arrest (I46) and respiratory failure, not elsewhere classified (J96), are other examples.
4. Unspecified causes within a larger cause grouping. For many diseases, such as neoplasms, a code is included within the grouping for an unspecified site. This is an illustration of a GC that is not important for assessing aggregate deaths from neoplasms from all sites but is important when assessing site-specific death rates. Another important example is the injury category in which some injuries are coded to unspecified factors or intent.

**Redistributing Deaths Assigned to GCs**

1. We carefully considered pathophysiology in identifying target causes for a GC. For example, for peritonitis, our targets include digestive diseases, such as intestinal obstruction; genitourinary diseases such as salpingitis and oophoritis; pregnancy, childbirth, and puerperium disease; conditions such as abortions; and some intentional/unintentional injuries.
2. We distinguished three methods for assigning GC deaths to a set of target underlying causes: proportionate redistribution within an age-sex group, statistical models, and expert judgment. We used a combination of all of these approaches depending on the four types of GCs. For causes with little information content, we used proportionate redistribution across target causes. In the case of heart failure, we developed a statistical model that helps identify the proportion of deaths for each target code within a given age-sex group. The algorithm eliminates all deaths with the code HF (ICD-10 I50) from the database. It identifies the fraction that should be extracted from HF and assigned to each of the target categories. To estimate the fractions allocated to each target code, we regressed by age, sex, and development status using all available ICD-10 mortality data the fraction of heart failure deaths from all deaths related to heart failure, including target causes.
3. For many GCs, we reviewed the published literature and engaged in consultation with GBD expert groups to develop an expert-based algorithm for assigning the fraction of deaths assigned to a GC within an age-sex group to be allocated to different target causes. A further criterion used in developing these expert algorithms was to compare the time trends in a cause by country across various revisions of the ICD. For example, the distribution of GCs to target codes for heart failure is a function of local epidemiology. Redistribution of GCs should in principle generate more plausible or continuous time trends commensurate with the underlying nature of a cause without observing the major discontinuities associated with a change in ICD.

**Step 5: Noise reduction**

To deal with problems of zero counts in vital registration, verbal autopsy, cancer registries, or sibling histories for a given age group in a given year, we use a Bayesian noise-reduction algorithm. For this algorithm, we assume a normal prior and a normal data likelihood. We estimate the normal prior for a given country-series of data by estimating a negative binomial for the fraction of deaths in each age group due to each respective cause with dummy variables for age and year.

**Step 6: Causes of death modelling methods**

1. In the Cause of death ensemble modelling (CODEm) framework, four families of statistical models are used: linear mixed effects regression (LMER) models of the natural log of the cause-specific death rate, LMER models of the logit of the cause fraction, spatiotemporal Gaussian process regression (ST-GPR) models of the natural logarithm of the cause-specific death rate, and ST-GPR models of the logit of the cause fraction. For each family of models, all plausible relationships between covariates and the response variable are identified. Because all possible combinations of selected covariates are considered for each family of models, multicollinearity between covariates may produce implausible signs on coefficients or unstable coefficients. Each combination is therefore tested for statistical significance (covariate coefficients must have a coefficient with p-value < 0∙05) and plausibility (the coefficients must have the directions expected based on the literature). Only covariate combinations meeting these criteria are retained. This selection process is run for both cause fractions and death rates, then ST-GPR and LMER-only models are created for each set of covariates.
2. For ten rare causes of death, there were too few observed deaths in the cause of death database to produce stable estimates. For these causes, we ran negative binomial regression models with either a constant or constant multiplied by the mean assumption for the dispersion parameter, using reverse step-wise model building. We selected between the two model dispersion assumptions on the basis of best fit to the data

**REFERENCE**

*[1] Naghavi M, Makela S, Foreman K, et al. Algorithms for enhancing public health utility of national causes-of-death data. Popul Health Metr. 2010; 8: 9. DOI: 10.1186/1478-7954-8-9*

*[2] Supplementary appendix 1: GBD 2016 Causes of Death Collaborators. Global, regional, and national age-sex specific mortality for 264 causes of death, 1980–2016: a systematic analysis for the Global Burden of Disease Study 2016. Lancet 2017; 390: 1151–210.*

*[3] Supplementary appendix: Zhou M, Wang H, Zhu J, et al. Cause-specific mortality for 240 causes in China during 1990–2013: a systematic subnational analysis for the Global Burden of Disease Study 2013. Lancet 2015; published online Oct 26. http://dx.doi.org/10.1016/S0140-6736 (15)00551-6.*

**Section 2: Detailed contributions stratified by age, cause and sex to life expectancy gap.**

**Table S4a** Number of years contributed by ages and causes to the life expectancy gap, China, 1990-2002

| Causes of death | Age groups | | | | | | | | | | | | | | | | | | |
| --- | --- | --- | --- | --- | --- | --- | --- | --- | --- | --- | --- | --- | --- | --- | --- | --- | --- | --- | --- |
|  | <1 | 1-4 | 5-9 | 10-14 | 15-19 | 20-24 | 25-29 | 30-34 | 35-39 | 40-44 | 45-49 | 50-54 | 55-59 | 60-64 | 65-69 | 70-74 | 75-79 | 80-84 | 85+ |
| A | 1.1359 | 0.2478 | 0.0356 | 0.0178 | 0.0156 | 0.0203 | 0.0147 | 0.0168 | 0.0156 | 0.0176 | 0.0215 | 0.0239 | 0.0269 | 0.0296 | 0.0289 | 0.0274 | 0.0182 | 0.0100 | 0.0052 |
| A1 | 0.0033 | 0.0049 | 0.0024 | 0.0031 | 0.0039 | 0.0055 | 0.0035 | 0.0049 | 0.0059 | 0.0085 | 0.0118 | 0.0147 | 0.0176 | 0.0198 | 0.0189 | 0.0166 | 0.0105 | 0.0049 | 0.0024 |
| A2 | 0.6535 | 0.2294 | 0.0277 | 0.0112 | 0.0064 | 0.0043 | 0.0032 | 0.0036 | 0.0032 | 0.0040 | 0.0048 | 0.0053 | 0.0058 | 0.0067 | 0.0072 | 0.0080 | 0.0058 | 0.0039 | 0.0018 |
| A3 | 0.0016 | 0.0040 | 0.0025 | 0.0014 | 0.0006 | 0.0005 | 0.0003 | 0.0004 | 0.0005 | 0.0006 | 0.0008 | 0.0008 | 0.0009 | 0.0007 | 0.0006 | 0.0004 | 0.0002 | 0.0001 | 0.0000 |
| A4 | 0.0000 | 0.0000 | 0.0000 | 0.0004 | 0.0030 | 0.0082 | 0.0059 | 0.0054 | 0.0033 | 0.0015 | 0.0006 | 0.0001 | 0.0000 | 0.0000 | 0.0000 | 0.0000 | 0.0000 | 0.0000 | 0.0000 |
| A5 | 0.4304 | -0.0008 | 0.0000 | 0.0000 | 0.0000 | 0.0000 | 0.0000 | 0.0000 | 0.0000 | 0.0000 | 0.0000 | 0.0000 | 0.0000 | 0.0000 | 0.0000 | 0.0000 | 0.0000 | 0.0000 | 0.0000 |
| A6 | 0.0350 | 0.0061 | 0.0015 | 0.0008 | 0.0006 | 0.0005 | 0.0003 | 0.0004 | 0.0004 | 0.0004 | 0.0005 | 0.0005 | 0.0006 | 0.0008 | 0.0008 | 0.0011 | 0.0010 | 0.0009 | 0.0009 |
| A7 | 0.0121 | 0.0043 | 0.0016 | 0.0010 | 0.0011 | 0.0014 | 0.0015 | 0.0021 | 0.0023 | 0.0027 | 0.0030 | 0.0025 | 0.0020 | 0.0015 | 0.0015 | 0.0012 | 0.0006 | 0.0002 | 0.0001 |
| B | 0.0745 | 0.0717 | 0.0278 | 0.0234 | 0.0328 | 0.0325 | 0.0175 | 0.0240 | 0.0218 | 0.0327 | 0.0771 | 0.1188 | 0.1735 | 0.2066 | 0.2007 | 0.1809 | 0.0499 | 0.0101 | 0.0098 |
| B1 | 0.0059 | 0.0105 | 0.0081 | 0.0061 | 0.0063 | 0.0056 | 0.0034 | 0.0068 | 0.0063 | 0.0100 | 0.0215 | 0.0227 | 0.0345 | 0.0303 | 0.0155 | -0.0054 | -0.0222 | -0.0142 | -0.0056 |
| B2 | 0.0154 | 0.0127 | 0.0062 | 0.0063 | 0.0114 | 0.0123 | 0.0041 | 0.0043 | 0.0021 | 0.0018 | 0.0185 | 0.0413 | 0.0650 | 0.0729 | 0.0693 | 0.0553 | -0.0061 | -0.0160 | -0.0139 |
| B3 | 0.0000 | 0.0064 | 0.0023 | 0.0022 | 0.0041 | 0.0041 | 0.0039 | 0.0060 | 0.0078 | 0.0124 | 0.0216 | 0.0373 | 0.0567 | 0.0889 | 0.1057 | 0.1258 | 0.0785 | 0.0408 | 0.0287 |
| B4 | 0.0000 | 0.0017 | 0.0007 | 0.0007 | 0.0009 | 0.0012 | 0.0012 | 0.0021 | 0.0033 | 0.0053 | 0.0090 | 0.0101 | 0.0087 | 0.0072 | 0.0060 | 0.0036 | 0.0012 | 0.0005 | 0.0002 |
| B5 | 0.0130 | 0.0156 | 0.0028 | 0.0018 | 0.0017 | 0.0019 | 0.0017 | 0.0023 | 0.0024 | 0.0034 | 0.0052 | 0.0065 | 0.0076 | 0.0081 | 0.0074 | 0.0069 | 0.0047 | 0.0025 | 0.0015 |
| B6 | 0.0020 | 0.0021 | 0.0014 | 0.0018 | 0.0019 | 0.0016 | 0.0010 | 0.0008 | 0.0005 | 0.0004 | 0.0004 | 0.0001 | -0.0002 | -0.0009 | -0.0012 | -0.0016 | -0.0011 | -0.0001 | 0.0007 |
| B7 | 0.0000 | 0.0000 | 0.0000 | 0.0000 | 0.0031 | 0.0036 | 0.0025 | 0.0017 | 0.0006 | 0.0002 | 0.0005 | 0.0005 | 0.0003 | 0.0007 | 0.0004 | 0.0005 | 0.0002 | 0.0000 | 0.0001 |
| B8 | 0.0094 | 0.0086 | 0.0036 | 0.0031 | 0.0026 | 0.0021 | 0.0003 | 0.0004 | -0.0008 | -0.0005 | 0.0005 | 0.0003 | 0.0009 | -0.0006 | -0.0025 | -0.0041 | -0.0053 | -0.0034 | -0.0018 |
| B9 | 0.0000 | 0.0000 | 0.0002 | 0.0003 | 0.0004 | 0.0003 | -0.0001 | 0.0000 | -0.0001 | 0.0000 | 0.0001 | 0.0001 | 0.0001 | 0.0001 | 0.0001 | 0.0001 | 0.0000 | 0.0000 | -0.0001 |
| B10 | 0.0289 | 0.0141 | 0.0025 | 0.0011 | 0.0005 | -0.0003 | -0.0005 | -0.0004 | -0.0003 | -0.0003 | -0.0001 | -0.0001 | -0.0001 | -0.0001 | -0.0001 | -0.0001 | -0.0001 | -0.0001 | -0.0001 |
| C | 0.0565 | 0.1509 | 0.0620 | 0.0422 | 0.0462 | 0.0398 | 0.0156 | 0.0075 | 0.0010 | 0.0023 | 0.0098 | 0.0087 | 0.0086 | 0.0067 | 0.0056 | 0.0041 | 0.0002 | -0.0002 | -0.0005 |
| C1 | 0.0001 | 0.0002 | 0.0002 | 0.0002 | 0.0010 | 0.0030 | 0.0030 | 0.0021 | 0.0014 | 0.0005 | 0.0007 | 0.0004 | 0.0002 | 0.0001 | 0.0001 | 0.0000 | 0.0000 | 0.0000 | 0.0000 |
| C2 | 0.0056 | 0.0045 | 0.0027 | 0.0091 | 0.0267 | 0.0398 | 0.0270 | 0.0210 | 0.0153 | 0.0123 | 0.0117 | 0.0093 | 0.0076 | 0.0061 | 0.0050 | 0.0038 | 0.0014 | 0.0006 | 0.0001 |
| C3 | 0.0064 | 0.0214 | 0.0104 | 0.0046 | 0.0009 | -0.0142 | -0.0194 | -0.0196 | -0.0184 | -0.0144 | -0.0084 | -0.0058 | -0.0032 | -0.0030 | -0.0024 | -0.0017 | -0.0018 | -0.0010 | -0.0006 |
| C4 | 0.0444 | 0.1248 | 0.0488 | 0.0283 | 0.0176 | 0.0112 | 0.0050 | 0.0041 | 0.0026 | 0.0040 | 0.0059 | 0.0048 | 0.0041 | 0.0035 | 0.0030 | 0.0020 | 0.0006 | 0.0002 | -0.0001 |
| TE | 1.2669 | 0.4705 | 0.1255 | 0.0834 | 0.0947 | 0.0927 | 0.0478 | 0.0483 | 0.0383 | 0.0527 | 0.1085 | 0.1515 | 0.2090 | 0.2429 | 0.2353 | 0.2124 | 0.0683 | 0.0198 | 0.0144 |

*Note: TE, total effect to the life expectancy gap. Gray shadow represents negative effect to the life expectancy gap; Value of ‘0.0000’ represents |effect|<0.0001.*

**Table S4b** Number of years contributed by ages and causes to the life expectancy gap, China, 2002-2007

| Causes of death | Age groups | | | | | | | | | | | | | | | | | | |
| --- | --- | --- | --- | --- | --- | --- | --- | --- | --- | --- | --- | --- | --- | --- | --- | --- | --- | --- | --- |
|  | <1 | 1-4 | 5-9 | 10-14 | 15-19 | 20-24 | 25-29 | 30-34 | 35-39 | 40-44 | 45-49 | 50-54 | 55-59 | 60-64 | 65-69 | 70-74 | 75-79 | 80-84 | 85+ |
| A | 0.4615 | 0.0528 | 0.0113 | 0.0055 | 0.0044 | 0.0064 | 0.0076 | 0.0073 | 0.0068 | 0.0053 | 0.0069 | 0.0072 | 0.0089 | 0.0136 | 0.0133 | 0.0144 | 0.0143 | 0.0100 | 0.0063 |
| A1 | 0.0010 | 0.0007 | 0.0006 | 0.0008 | 0.0011 | 0.0015 | 0.0015 | 0.0016 | 0.0016 | 0.0017 | 0.0029 | 0.0035 | 0.0050 | 0.0078 | 0.0073 | 0.0071 | 0.0060 | 0.0032 | 0.0014 |
| A2 | 0.2162 | 0.0491 | 0.0092 | 0.0036 | 0.0019 | 0.0018 | 0.0019 | 0.0018 | 0.0015 | 0.0012 | 0.0016 | 0.0015 | 0.0020 | 0.0036 | 0.0039 | 0.0055 | 0.0067 | 0.0058 | 0.0041 |
| A3 | 0.0006 | 0.0013 | 0.0007 | 0.0004 | 0.0002 | 0.0002 | 0.0002 | 0.0003 | 0.0003 | 0.0003 | 0.0003 | 0.0004 | 0.0004 | 0.0004 | 0.0003 | 0.0002 | 0.0001 | 0.0001 | 0.0000 |
| A4 | 0.0000 | 0.0000 | 0.0000 | 0.0001 | 0.0007 | 0.0021 | 0.0030 | 0.0022 | 0.0018 | 0.0005 | 0.0001 | 0.0000 | 0.0000 | 0.0000 | 0.0000 | 0.0000 | 0.0000 | 0.0000 | 0.0000 |
| A5 | 0.2305 | 0.0003 | 0.0000 | 0.0000 | 0.0000 | 0.0000 | 0.0000 | 0.0000 | 0.0000 | 0.0000 | 0.0000 | 0.0000 | 0.0000 | 0.0000 | 0.0000 | 0.0000 | 0.0000 | 0.0000 | 0.0000 |
| A6 | 0.0097 | 0.0003 | 0.0003 | 0.0002 | 0.0001 | 0.0002 | 0.0002 | 0.0002 | 0.0002 | 0.0001 | 0.0001 | 0.0001 | 0.0002 | 0.0002 | 0.0003 | 0.0004 | 0.0007 | 0.0006 | 0.0005 |
| A7 | 0.0035 | 0.0011 | 0.0005 | 0.0004 | 0.0004 | 0.0007 | 0.0009 | 0.0014 | 0.0016 | 0.0015 | 0.0019 | 0.0017 | 0.0015 | 0.0017 | 0.0016 | 0.0011 | 0.0008 | 0.0004 | 0.0002 |
| B | 0.1258 | 0.0315 | 0.0178 | 0.0163 | 0.0142 | 0.0225 | 0.0334 | 0.0439 | 0.0574 | 0.0513 | 0.0934 | 0.0803 | 0.1073 | 0.2303 | 0.2243 | 0.2673 | 0.2830 | 0.1536 | 0.0735 |
| B1 | 0.0037 | 0.0063 | 0.0061 | 0.0056 | 0.0047 | 0.0056 | 0.0097 | 0.0155 | 0.0228 | 0.0227 | 0.0387 | 0.0294 | 0.0311 | 0.0584 | 0.0428 | 0.0395 | 0.0304 | 0.0087 | 0.0035 |
| B2 | 0.0126 | 0.0044 | 0.0030 | 0.0030 | 0.0031 | 0.0063 | 0.0093 | 0.0117 | 0.0160 | 0.0131 | 0.0298 | 0.0238 | 0.0372 | 0.0951 | 0.0930 | 0.1065 | 0.1222 | 0.0599 | 0.0195 |
| B3 | 0.0000 | 0.0008 | 0.0005 | 0.0005 | 0.0008 | 0.0016 | 0.0025 | 0.0034 | 0.0044 | 0.0054 | 0.0099 | 0.0154 | 0.0254 | 0.0546 | 0.0701 | 0.1043 | 0.1138 | 0.0772 | 0.0469 |
| B4 | 0.0000 | 0.0006 | 0.0004 | 0.0004 | 0.0004 | 0.0010 | 0.0021 | 0.0035 | 0.0046 | 0.0042 | 0.0066 | 0.0055 | 0.0062 | 0.0082 | 0.0059 | 0.0050 | 0.0042 | 0.0019 | 0.0007 |
| B5 | -0.0001 | -0.0001 | 0.0008 | 0.0006 | 0.0005 | 0.0009 | 0.0013 | 0.0016 | 0.0017 | 0.0015 | 0.0024 | 0.0023 | 0.0031 | 0.0049 | 0.0047 | 0.0052 | 0.0052 | 0.0032 | 0.0019 |
| B6 | 0.0016 | 0.0013 | 0.0010 | 0.0012 | 0.0009 | 0.0013 | 0.0012 | 0.0012 | 0.0009 | 0.0005 | 0.0006 | 0.0004 | 0.0002 | 0.0001 | -0.0004 | -0.0008 | -0.0008 | -0.0007 | -0.0009 |
| B7 | 0.0000 | 0.0000 | 0.0000 | 0.0000 | 0.0012 | 0.0022 | 0.0025 | 0.0021 | 0.0015 | 0.0007 | 0.0006 | 0.0004 | 0.0005 | 0.0006 | 0.0003 | 0.0003 | 0.0003 | 0.0001 | 0.0001 |
| B8 | 0.0067 | 0.0037 | 0.0020 | 0.0020 | 0.0015 | 0.0026 | 0.0037 | 0.0042 | 0.0046 | 0.0030 | 0.0045 | 0.0031 | 0.0035 | 0.0082 | 0.0074 | 0.0069 | 0.0075 | 0.0032 | 0.0017 |
| B9 | 0.0000 | 0.0000 | 0.0002 | 0.0003 | 0.0003 | 0.0004 | 0.0005 | 0.0003 | 0.0004 | 0.0002 | 0.0002 | 0.0002 | 0.0001 | 0.0003 | 0.0003 | 0.0003 | 0.0003 | 0.0001 | 0.0001 |
| B10 | 0.1013 | 0.0144 | 0.0038 | 0.0028 | 0.0007 | 0.0007 | 0.0007 | 0.0004 | 0.0003 | 0.0001 | 0.0001 | 0.0000 | 0.0000 | 0.0000 | 0.0000 | 0.0000 | 0.0000 | 0.0000 | 0.0000 |
| C | 0.0255 | 0.0409 | 0.0383 | 0.0323 | 0.0124 | 0.0253 | 0.0306 | 0.0292 | 0.0190 | -0.0008 | 0.0075 | -0.0010 | -0.0013 | 0.0043 | 0.0028 | 0.0040 | 0.0048 | 0.0016 | -0.0006 |
| C1 | 0.0000 | 0.0000 | 0.0000 | 0.0000 | 0.0002 | 0.0011 | 0.0010 | 0.0009 | 0.0008 | 0.0004 | 0.0002 | 0.0001 | 0.0001 | 0.0000 | 0.0000 | 0.0000 | 0.0000 | 0.0000 | 0.0000 |
| C2 | 0.0028 | 0.0023 | 0.0020 | 0.0044 | 0.0070 | 0.0127 | 0.0152 | 0.0132 | 0.0102 | 0.0052 | 0.0058 | 0.0033 | 0.0023 | 0.0029 | 0.0018 | 0.0020 | 0.0018 | 0.0008 | 0.0002 |
| C3 | 0.0017 | 0.0040 | 0.0108 | 0.0076 | 0.0016 | 0.0083 | 0.0104 | 0.0118 | 0.0080 | -0.0026 | 0.0020 | -0.0027 | -0.0025 | 0.0002 | 0.0004 | 0.0013 | 0.0018 | 0.0007 | 0.0002 |
| C4 | 0.0211 | 0.0346 | 0.0255 | 0.0202 | 0.0037 | 0.0032 | 0.0040 | 0.0032 | 0.0000 | -0.0038 | -0.0005 | -0.0018 | -0.0012 | 0.0011 | 0.0005 | 0.0008 | 0.0011 | 0.0002 | -0.0010 |
| TE | 0.6128 | 0.1252 | 0.0674 | 0.0540 | 0.0310 | 0.0542 | 0.0716 | 0.0805 | 0.0832 | 0.0557 | 0.1078 | 0.0865 | 0.1149 | 0.2482 | 0.2403 | 0.2858 | 0.3021 | 0.1653 | 0.0791 |

*Note: TE, total effect to the life expectancy gap. Gray shadow represents negative effect to the life expectancy gap; Value of ‘0.0000’ represents |effect|<0.0001.*

**Table S4c** Number of years contributed by ages and causes to the life expectancy gap, China, 2007-2016

| Causes of death | Age groups | | | | | | | | | | | | | | | | | | |
| --- | --- | --- | --- | --- | --- | --- | --- | --- | --- | --- | --- | --- | --- | --- | --- | --- | --- | --- | --- |
|  | <1 | 1-4 | 5-9 | 10-14 | 15-19 | 20-24 | 25-29 | 30-34 | 35-39 | 40-44 | 45-49 | 50-54 | 55-59 | 60-64 | 65-69 | 70-74 | 75-79 | 80-84 | 85+ |
| A | 0.4279 | 0.0345 | 0.0066 | 0.0024 | 0.0038 | 0.0061 | 0.0046 | 0.0039 | 0.0043 | 0.0048 | 0.0044 | 0.0071 | 0.0091 | 0.0084 | 0.0102 | 0.0127 | 0.0111 | 0.0103 | 0.0103 |
| A1 | 0.0004 | 0.0004 | 0.0001 | 0.0000 | 0.0011 | 0.0021 | 0.0012 | 0.0003 | 0.0007 | 0.0015 | 0.0019 | 0.0036 | 0.0050 | 0.0048 | 0.0052 | 0.0057 | 0.0041 | 0.0026 | 0.0015 |
| A2 | 0.1331 | 0.0315 | 0.0052 | 0.0015 | 0.0016 | 0.0016 | 0.0010 | 0.0011 | 0.0013 | 0.0015 | 0.0012 | 0.0018 | 0.0024 | 0.0023 | 0.0036 | 0.0058 | 0.0060 | 0.0070 | 0.0083 |
| A3 | 0.0003 | 0.0009 | 0.0010 | 0.0006 | 0.0003 | 0.0002 | 0.0002 | 0.0002 | 0.0003 | 0.0004 | 0.0004 | 0.0004 | 0.0005 | 0.0004 | 0.0004 | 0.0003 | 0.0002 | 0.0001 | 0.0001 |
| A4 | 0.0000 | 0.0000 | 0.0000 | 0.0000 | 0.0006 | 0.0018 | 0.0018 | 0.0016 | 0.0011 | 0.0004 | 0.0001 | 0.0000 | 0.0000 | 0.0000 | 0.0000 | 0.0000 | 0.0000 | 0.0000 | 0.0000 |
| A5 | 0.2877 | 0.0009 | 0.0000 | 0.0000 | 0.0000 | 0.0000 | 0.0000 | 0.0000 | 0.0000 | 0.0000 | 0.0000 | 0.0000 | 0.0000 | 0.0000 | 0.0000 | 0.0000 | 0.0000 | 0.0000 | 0.0000 |
| A6 | 0.0044 | 0.0002 | 0.0001 | 0.0000 | 0.0001 | 0.0001 | 0.0001 | 0.0001 | 0.0001 | 0.0001 | 0.0001 | 0.0001 | 0.0001 | 0.0001 | 0.0001 | 0.0002 | 0.0002 | 0.0003 | 0.0003 |
| A7 | 0.0019 | 0.0006 | 0.0002 | 0.0001 | 0.0002 | 0.0003 | 0.0003 | 0.0006 | 0.0008 | 0.0009 | 0.0008 | 0.0011 | 0.0012 | 0.0008 | 0.0009 | 0.0007 | 0.0005 | 0.0003 | 0.0002 |
| B | 0.1152 | 0.0317 | 0.0089 | 0.0068 | 0.0141 | 0.0184 | 0.0125 | 0.0253 | 0.0556 | 0.0952 | 0.0747 | 0.1380 | 0.1857 | 0.1385 | 0.2176 | 0.2943 | 0.2314 | 0.1688 | 0.1147 |
| B1 | 0.0016 | 0.0048 | 0.0020 | 0.0023 | 0.0045 | 0.0045 | 0.0035 | 0.0089 | 0.0217 | 0.0378 | 0.0334 | 0.0554 | 0.0659 | 0.0361 | 0.0531 | 0.0611 | 0.0365 | 0.0145 | 0.0072 |
| B2 | 0.0135 | 0.0052 | 0.0020 | 0.0015 | 0.0033 | 0.0051 | 0.0017 | 0.0060 | 0.0173 | 0.0341 | 0.0225 | 0.0501 | 0.0736 | 0.0561 | 0.0930 | 0.1251 | 0.0972 | 0.0719 | 0.0439 |
| B3 | 0.0000 | 0.0005 | 0.0003 | 0.0002 | 0.0007 | 0.0011 | 0.0013 | 0.0019 | 0.0032 | 0.0055 | 0.0065 | 0.0142 | 0.0241 | 0.0333 | 0.0553 | 0.0891 | 0.0858 | 0.0747 | 0.0593 |
| B4 | 0.0000 | 0.0005 | 0.0002 | 0.0002 | 0.0004 | 0.0009 | 0.0012 | 0.0023 | 0.0044 | 0.0066 | 0.0049 | 0.0069 | 0.0078 | 0.0045 | 0.0049 | 0.0050 | 0.0031 | 0.0018 | 0.0009 |
| B5 | -0.0001 | 0.0008 | 0.0005 | 0.0003 | 0.0005 | 0.0008 | 0.0008 | 0.0011 | 0.0015 | 0.0020 | 0.0019 | 0.0028 | 0.0037 | 0.0034 | 0.0045 | 0.0059 | 0.0051 | 0.0042 | 0.0033 |
| B6 | 0.0007 | 0.0010 | 0.0005 | 0.0003 | 0.0007 | 0.0008 | 0.0005 | 0.0007 | 0.0008 | 0.0007 | 0.0004 | 0.0006 | 0.0005 | -0.0002 | -0.0003 | -0.0005 | -0.0015 | 0.0786 | -0.0006 |
| B7 | 0.0000 | 0.0000 | 0.0000 | 0.0000 | 0.0004 | 0.0009 | 0.0007 | 0.0009 | 0.0011 | 0.0013 | 0.0008 | 0.0009 | 0.0007 | -0.0001 | -0.0001 | -0.0002 | -0.0003 | -0.0002 | -0.0001 |
| B8 | 0.0034 | 0.0030 | 0.0011 | 0.0008 | 0.0018 | 0.0027 | 0.0022 | 0.0030 | 0.0048 | 0.0063 | 0.0041 | 0.0065 | 0.0089 | 0.0053 | 0.0068 | 0.0083 | 0.0049 | 0.0018 | 0.0005 |
| B9 | 0.0000 | 0.0000 | 0.0001 | 0.0001 | 0.0005 | 0.0005 | 0.0002 | 0.0002 | 0.0003 | 0.0004 | 0.0002 | 0.0003 | 0.0004 | 0.0002 | 0.0003 | 0.0004 | 0.0003 | 0.0002 | 0.0001 |
| B10 | 0.0962 | 0.0160 | 0.0023 | 0.0013 | 0.0013 | 0.0011 | 0.0004 | 0.0003 | 0.0004 | 0.0003 | 0.0001 | 0.0001 | 0.0001 | 0.0001 | 0.0001 | 0.0001 | 0.0001 | 0.0002 | 0.0002 |
| C | 0.0206 | 0.0515 | 0.0321 | 0.0186 | 0.0303 | 0.0448 | 0.0301 | 0.0387 | 0.0489 | 0.0449 | 0.0211 | 0.0240 | 0.0196 | 0.0073 | 0.0100 | 0.0123 | 0.0096 | 0.0072 | 0.0048 |
| C1 | 0.0000 | 0.0000 | 0.0000 | 0.0000 | 0.0001 | 0.0004 | 0.0002 | 0.0003 | 0.0002 | 0.0002 | 0.0001 | 0.0001 | 0.0000 | 0.0000 | 0.0000 | 0.0000 | 0.0000 | 0.0000 | 0.0000 |
| C2 | 0.0018 | 0.0017 | 0.0010 | 0.0023 | 0.0075 | 0.0111 | 0.0089 | 0.0098 | 0.0112 | 0.0101 | 0.0070 | 0.0084 | 0.0076 | 0.0051 | 0.0057 | 0.0061 | 0.0047 | 0.0032 | 0.0016 |
| C3 | 0.0006 | 0.0094 | 0.0099 | 0.0042 | 0.0141 | 0.0232 | 0.0144 | 0.0192 | 0.0249 | 0.0233 | 0.0091 | 0.0097 | 0.0071 | 0.0010 | 0.0023 | 0.0029 | 0.0021 | 0.0012 | 0.0007 |
| C4 | 0.0182 | 0.0404 | 0.0212 | 0.0121 | 0.0087 | 0.0101 | 0.0065 | 0.0094 | 0.0127 | 0.0113 | 0.0049 | 0.0059 | 0.0048 | 0.0012 | 0.0020 | 0.0033 | 0.0028 | 0.0029 | 0.0025 |
| TE | 0.5636 | 0.1177 | 0.0476 | 0.0277 | 0.0482 | 0.0692 | 0.0472 | 0.0678 | 0.1088 | 0.1449 | 0.1003 | 0.1691 | 0.2144 | 0.1542 | 0.2378 | 0.3193 | 0.2521 | 0.1862 | 0.1298 |

*Note: TE, total effect to the life expectancy gap. Gray shadow represents negative effect to the life expectancy gap; Value of ‘0.0000’ represents |effect|<0.0001.*

**Table S5a** Number of years contributed by ages and causes to the life expectancy gap for male, China, 1990-2002.

| Causes of death | Age groups | | | | | | | | | | | | | | | | | | |
| --- | --- | --- | --- | --- | --- | --- | --- | --- | --- | --- | --- | --- | --- | --- | --- | --- | --- | --- | --- |
|  | <1 | 1-4 | 5-9 | 10-14 | 15-19 | 20-24 | 25-29 | 30-34 | 35-39 | 40-44 | 45-49 | 50-54 | 55-59 | 60-64 | 65-69 | 70-74 | 75-79 | 80-84 | 85+ |
| A | 1.1002 | 0.2059 | 0.0310 | 0.0151 | 0.0123 | 0.0116 | 0.0085 | 0.0105 | 0.0120 | 0.0173 | 0.0226 | 0.0259 | 0.0291 | 0.0317 | 0.0299 | 0.0245 | 0.0141 | 0.0065 | 0.0028 |
| A1 | 0.0041 | 0.0036 | 0.0026 | 0.0026 | 0.0039 | 0.0052 | 0.0030 | 0.0037 | 0.0050 | 0.0083 | 0.0121 | 0.0162 | 0.0198 | 0.0225 | 0.0211 | 0.0160 | 0.0095 | 0.0042 | 0.0019 |
| A2 | 0.6064 | 0.1919 | 0.0240 | 0.0100 | 0.0062 | 0.0040 | 0.0029 | 0.0034 | 0.0030 | 0.0042 | 0.0050 | 0.0053 | 0.0056 | 0.0064 | 0.0063 | 0.0063 | 0.0035 | 0.0017 | 0.0005 |
| A3 | 0.0013 | 0.0026 | 0.0015 | 0.0009 | 0.0006 | 0.0004 | 0.0003 | 0.0004 | 0.0005 | 0.0006 | 0.0008 | 0.0008 | 0.0009 | 0.0007 | 0.0005 | 0.0003 | 0.0001 | 0.0000 | 0.0000 |
| A4 | 0.0000 | 0.0000 | 0.0000 | 0.0000 | 0.0000 | 0.0000 | 0.0000 | 0.0000 | 0.0000 | 0.0000 | 0.0000 | 0.0000 | 0.0000 | 0.0000 | 0.0000 | 0.0000 | 0.0000 | 0.0000 | 0.0000 |
| A5 | 0.4568 | -0.0007 | 0.0000 | 0.0000 | 0.0000 | 0.0000 | 0.0000 | 0.0000 | 0.0000 | 0.0000 | 0.0000 | 0.0000 | 0.0000 | 0.0000 | 0.0000 | 0.0000 | 0.0000 | 0.0000 | 0.0000 |
| A6 | 0.0164 | 0.0040 | 0.0013 | 0.0007 | 0.0006 | 0.0004 | 0.0003 | 0.0003 | 0.0003 | 0.0003 | 0.0005 | 0.0005 | 0.0006 | 0.0007 | 0.0006 | 0.0007 | 0.0005 | 0.0004 | 0.0003 |
| A7 | 0.0152 | 0.0046 | 0.0016 | 0.0010 | 0.0011 | 0.0016 | 0.0019 | 0.0027 | 0.0032 | 0.0039 | 0.0042 | 0.0031 | 0.0022 | 0.0015 | 0.0013 | 0.0012 | 0.0004 | 0.0002 | 0.0001 |
| B | 0.0794 | 0.0547 | 0.0247 | 0.0198 | 0.0311 | 0.0278 | 0.0150 | 0.0108 | 0.0141 | 0.0189 | 0.0553 | 0.0990 | 0.1521 | 0.1957 | 0.1852 | 0.1420 | -0.0006 | -0.0143 | -0.0031 |
| B1 | 0.0053 | 0.0075 | 0.0072 | 0.0049 | 0.0055 | 0.0044 | 0.0031 | 0.0031 | 0.0061 | 0.0092 | 0.0167 | 0.0174 | 0.0293 | 0.0293 | 0.0138 | -0.0123 | -0.0280 | -0.0146 | -0.0048 |
| B2 | 0.0141 | 0.0097 | 0.0056 | 0.0056 | 0.0120 | 0.0111 | 0.0030 | -0.0021 | -0.0038 | -0.0107 | 0.0023 | 0.0264 | 0.0477 | 0.0584 | 0.0566 | 0.0370 | -0.0277 | -0.0261 | -0.0150 |
| B3 | 0.0000 | 0.0052 | 0.0024 | 0.0022 | 0.0040 | 0.0045 | 0.0037 | 0.0049 | 0.0067 | 0.0113 | 0.0205 | 0.0375 | 0.0594 | 0.0934 | 0.1054 | 0.1137 | 0.0585 | 0.0292 | 0.0183 |
| B4 | 0.0000 | 0.0011 | 0.0006 | 0.0005 | 0.0008 | 0.0010 | 0.0015 | 0.0026 | 0.0041 | 0.0064 | 0.0099 | 0.0102 | 0.0078 | 0.0070 | 0.0050 | 0.0025 | 0.0003 | 0.0000 | 0.0000 |
| B5 | 0.0134 | 0.0119 | 0.0027 | 0.0015 | 0.0017 | 0.0018 | 0.0018 | 0.0022 | 0.0027 | 0.0039 | 0.0061 | 0.0073 | 0.0084 | 0.0088 | 0.0074 | 0.0060 | 0.0036 | 0.0019 | 0.0010 |
| B6 | 0.0022 | 0.0023 | 0.0016 | 0.0021 | 0.0022 | 0.0016 | 0.0011 | 0.0009 | 0.0006 | 0.0005 | 0.0005 | 0.0002 | -0.0004 | -0.0010 | -0.0014 | -0.0021 | -0.0024 | -0.0018 | -0.0011 |
| B7 | 0.0000 | 0.0000 | 0.0000 | 0.0000 | 0.0020 | 0.0025 | 0.0013 | 0.0005 | -0.0008 | -0.0010 | -0.0005 | -0.0004 | -0.0002 | 0.0005 | 0.0001 | 0.0002 | 0.0000 | -0.0001 | 0.0000 |
| B8 | 0.0085 | 0.0067 | 0.0029 | 0.0023 | 0.0023 | 0.0013 | 0.0000 | -0.0008 | -0.0010 | -0.0006 | 0.0000 | 0.0003 | 0.0001 | -0.0007 | -0.0017 | -0.0031 | -0.0050 | -0.0029 | -0.0014 |
| B9 | 0.0000 | 0.0000 | 0.0002 | 0.0002 | 0.0002 | 0.0001 | 0.0000 | 0.0000 | 0.0000 | 0.0001 | 0.0001 | 0.0001 | 0.0001 | 0.0002 | 0.0001 | 0.0001 | 0.0000 | 0.0000 | 0.0000 |
| B10 | 0.0358 | 0.0102 | 0.0015 | 0.0005 | 0.0004 | -0.0007 | -0.0006 | -0.0006 | -0.0004 | -0.0003 | -0.0002 | -0.0001 | -0.0001 | -0.0001 | -0.0001 | -0.0001 | -0.0001 | -0.0001 | -0.0001 |
| C | 0.0468 | 0.1386 | 0.0696 | 0.0422 | 0.0417 | 0.0176 | 0.0008 | -0.0120 | -0.0121 | -0.0086 | 0.0009 | 0.0022 | 0.0041 | 0.0045 | 0.0037 | 0.0020 | -0.0019 | -0.0011 | -0.0007 |
| C1 | 0.0002 | 0.0003 | 0.0003 | 0.0003 | 0.0017 | 0.0056 | 0.0054 | 0.0038 | 0.0026 | 0.0008 | 0.0011 | 0.0007 | 0.0003 | 0.0002 | 0.0001 | 0.0001 | 0.0000 | 0.0000 | 0.0000 |
| C2 | 0.0040 | 0.0038 | 0.0020 | 0.0064 | 0.0173 | 0.0204 | 0.0143 | 0.0105 | 0.0086 | 0.0068 | 0.0077 | 0.0063 | 0.0051 | 0.0049 | 0.0037 | 0.0023 | 0.0002 | 0.0000 | 0.0000 |
| C3 | 0.0076 | 0.0199 | 0.0111 | 0.0040 | -0.0006 | -0.0233 | -0.0262 | -0.0300 | -0.0259 | -0.0205 | -0.0140 | -0.0096 | -0.0055 | -0.0040 | -0.0029 | -0.0017 | -0.0021 | -0.0011 | -0.0006 |
| C4 | 0.0350 | 0.1146 | 0.0561 | 0.0315 | 0.0233 | 0.0149 | 0.0073 | 0.0038 | 0.0027 | 0.0042 | 0.0061 | 0.0048 | 0.0041 | 0.0035 | 0.0027 | 0.0014 | 0.0001 | -0.0001 | -0.0002 |
| TE | 1.2263 | 0.3992 | 0.1252 | 0.0771 | 0.0851 | 0.0571 | 0.0243 | 0.0092 | 0.0140 | 0.0276 | 0.0788 | 0.1271 | 0.1852 | 0.2320 | 0.2187 | 0.1685 | 0.0117 | -0.0089 | -0.0011 |

*Note: TE, total effect to the life expectancy gap. Gray shadow represents negative effect to the life expectancy gap; Value of ‘0.0000’ represents |effect|<0.0001.*

**Table S5b** Number of years contributed by ages and causes to the life expectancy gap for female, China, 1990-2002.

| Causes of death | Age groups | | | | | | | | | | | | | | | | | | |
| --- | --- | --- | --- | --- | --- | --- | --- | --- | --- | --- | --- | --- | --- | --- | --- | --- | --- | --- | --- |
|  | <1 | 1-4 | 5-9 | 10-14 | 15-19 | 20-24 | 25-29 | 30-34 | 35-39 | 40-44 | 45-49 | 50-54 | 55-59 | 60-64 | 65-69 | 70-74 | 75-79 | 80-84 | 85+ |
| A | 1.1820 | 0.2971 | 0.0411 | 0.0209 | 0.0195 | 0.0304 | 0.0221 | 0.0244 | 0.0198 | 0.0180 | 0.0193 | 0.0200 | 0.0229 | 0.0258 | 0.0272 | 0.0306 | 0.0239 | 0.0151 | 0.0085 |
| A1 | 0.0024 | 0.0065 | 0.0022 | 0.0036 | 0.0039 | 0.0058 | 0.0041 | 0.0063 | 0.0069 | 0.0088 | 0.0109 | 0.0120 | 0.0140 | 0.0155 | 0.0154 | 0.0169 | 0.0120 | 0.0061 | 0.0031 |
| A2 | 0.7102 | 0.2733 | 0.0320 | 0.0126 | 0.0067 | 0.0046 | 0.0035 | 0.0039 | 0.0034 | 0.0037 | 0.0045 | 0.0050 | 0.0058 | 0.0071 | 0.0085 | 0.0103 | 0.0092 | 0.0070 | 0.0037 |
| A3 | 0.0019 | 0.0056 | 0.0036 | 0.0019 | 0.0005 | 0.0005 | 0.0003 | 0.0004 | 0.0005 | 0.0005 | 0.0006 | 0.0007 | 0.0008 | 0.0007 | 0.0007 | 0.0006 | 0.0003 | 0.0001 | 0.0000 |
| A4 | 0.0000 | 0.0000 | 0.0000 | 0.0009 | 0.0066 | 0.0177 | 0.0129 | 0.0120 | 0.0073 | 0.0033 | 0.0013 | 0.0003 | 0.0000 | 0.0000 | 0.0000 | 0.0000 | 0.0000 | 0.0000 | 0.0000 |
| A5 | 0.4028 | -0.0007 | 0.0000 | 0.0000 | 0.0000 | 0.0000 | 0.0000 | 0.0000 | 0.0000 | 0.0000 | 0.0000 | 0.0000 | 0.0000 | 0.0000 | 0.0000 | 0.0000 | 0.0000 | 0.0000 | 0.0000 |
| A6 | 0.0563 | 0.0085 | 0.0017 | 0.0010 | 0.0005 | 0.0005 | 0.0004 | 0.0005 | 0.0004 | 0.0005 | 0.0005 | 0.0005 | 0.0007 | 0.0009 | 0.0009 | 0.0015 | 0.0016 | 0.0016 | 0.0016 |
| A7 | 0.0084 | 0.0039 | 0.0017 | 0.0010 | 0.0012 | 0.0013 | 0.0010 | 0.0012 | 0.0013 | 0.0012 | 0.0014 | 0.0015 | 0.0017 | 0.0016 | 0.0017 | 0.0013 | 0.0008 | 0.0003 | 0.0001 |
| B | 0.0701 | 0.0911 | 0.0320 | 0.0279 | 0.0350 | 0.0383 | 0.0204 | 0.0392 | 0.0304 | 0.0492 | 0.0974 | 0.1299 | 0.1917 | 0.2205 | 0.2317 | 0.2396 | 0.1398 | 0.0614 | 0.0335 |
| B1 | 0.0065 | 0.0137 | 0.0093 | 0.0077 | 0.0073 | 0.0071 | 0.0036 | 0.0110 | 0.0063 | 0.0107 | 0.0241 | 0.0226 | 0.0369 | 0.0331 | 0.0248 | 0.0094 | -0.0063 | -0.0078 | -0.0043 |
| B2 | 0.0170 | 0.0163 | 0.0069 | 0.0072 | 0.0108 | 0.0137 | 0.0055 | 0.0116 | 0.0089 | 0.0170 | 0.0373 | 0.0570 | 0.0852 | 0.0929 | 0.0902 | 0.0833 | 0.0332 | 0.0055 | -0.0087 |
| B3 | 0.0000 | 0.0078 | 0.0022 | 0.0022 | 0.0043 | 0.0037 | 0.0041 | 0.0072 | 0.0092 | 0.0136 | 0.0227 | 0.0355 | 0.0509 | 0.0802 | 0.1050 | 0.1391 | 0.1083 | 0.0610 | 0.0434 |
| B4 | 0.0000 | 0.0025 | 0.0009 | 0.0009 | 0.0011 | 0.0014 | 0.0008 | 0.0014 | 0.0021 | 0.0037 | 0.0066 | 0.0082 | 0.0092 | 0.0075 | 0.0077 | 0.0053 | 0.0028 | 0.0013 | 0.0005 |
| B5 | 0.0124 | 0.0199 | 0.0030 | 0.0021 | 0.0017 | 0.0020 | 0.0016 | 0.0023 | 0.0021 | 0.0027 | 0.0037 | 0.0050 | 0.0063 | 0.0069 | 0.0074 | 0.0080 | 0.0063 | 0.0035 | 0.0022 |
| B6 | 0.0018 | 0.0019 | 0.0012 | 0.0014 | 0.0014 | 0.0016 | 0.0008 | 0.0008 | 0.0004 | 0.0003 | 0.0003 | 0.0000 | 0.0001 | -0.0007 | -0.0009 | -0.0008 | 0.0004 | 0.0014 | 0.0023 |
| B7 | 0.0000 | 0.0000 | 0.0000 | 0.0000 | 0.0044 | 0.0049 | 0.0039 | 0.0030 | 0.0023 | 0.0017 | 0.0016 | 0.0012 | 0.0010 | 0.0012 | 0.0011 | 0.0009 | 0.0006 | 0.0002 | 0.0002 |
| B8 | 0.0104 | 0.0107 | 0.0044 | 0.0041 | 0.0028 | 0.0032 | 0.0007 | 0.0019 | -0.0005 | -0.0003 | 0.0011 | 0.0004 | 0.0020 | -0.0005 | -0.0036 | -0.0054 | -0.0053 | -0.0035 | -0.0018 |
| B9 | 0.0000 | 0.0000 | 0.0003 | 0.0005 | 0.0006 | 0.0004 | -0.0002 | 0.0001 | -0.0002 | -0.0001 | 0.0001 | 0.0001 | 0.0001 | 0.0000 | 0.0000 | 0.0000 | -0.0001 | -0.0001 | -0.0001 |
| B10 | 0.0220 | 0.0183 | 0.0038 | 0.0018 | 0.0006 | 0.0002 | -0.0004 | -0.0001 | -0.0002 | -0.0002 | -0.0001 | -0.0001 | -0.0001 | -0.0001 | -0.0001 | -0.0001 | -0.0001 | -0.0002 | -0.0002 |
| C | 0.0684 | 0.1661 | 0.0562 | 0.0435 | 0.0518 | 0.0666 | 0.0332 | 0.0299 | 0.0160 | 0.0155 | 0.0189 | 0.0148 | 0.0139 | 0.0100 | 0.0089 | 0.0073 | 0.0036 | 0.0014 | -0.0001 |
| C1 | 0.0000 | 0.0001 | 0.0001 | 0.0001 | 0.0001 | 0.0001 | 0.0001 | 0.0000 | 0.0000 | 0.0000 | 0.0000 | 0.0000 | 0.0000 | 0.0000 | 0.0000 | 0.0000 | 0.0000 | 0.0000 | 0.0000 |
| C2 | 0.0074 | 0.0054 | 0.0034 | 0.0122 | 0.0377 | 0.0622 | 0.0419 | 0.0335 | 0.0234 | 0.0189 | 0.0167 | 0.0132 | 0.0107 | 0.0079 | 0.0067 | 0.0058 | 0.0031 | 0.0014 | 0.0003 |
| C3 | 0.0048 | 0.0229 | 0.0103 | 0.0055 | 0.0028 | -0.0029 | -0.0112 | -0.0078 | -0.0097 | -0.0070 | -0.0025 | -0.0020 | -0.0005 | -0.0014 | -0.0013 | -0.0013 | -0.0010 | -0.0007 | -0.0005 |
| C4 | 0.0562 | 0.1377 | 0.0424 | 0.0256 | 0.0113 | 0.0073 | 0.0024 | 0.0042 | 0.0023 | 0.0036 | 0.0046 | 0.0036 | 0.0036 | 0.0035 | 0.0034 | 0.0028 | 0.0015 | 0.0007 | 0.0001 |
| TE | 1.3205 | 0.5543 | 0.1293 | 0.0923 | 0.1063 | 0.1352 | 0.0757 | 0.0934 | 0.0663 | 0.0827 | 0.1356 | 0.1647 | 0.2284 | 0.2563 | 0.2678 | 0.2775 | 0.1673 | 0.0778 | 0.0419 |

*Note: TE, total effect to the life expectancy gap. Gray shadow represents negative effect to the life expectancy gap; Value of ‘0.0000’ represents |effect|<0.0001.*

**Table S6a** Number of years contributed by ages and causes to the life expectancy gap for male, China, 2002-2007.

| Causes of death | Age groups | | | | | | | | | | | | | | | | | | |
| --- | --- | --- | --- | --- | --- | --- | --- | --- | --- | --- | --- | --- | --- | --- | --- | --- | --- | --- | --- |
|  | <1 | 1-4 | 5-9 | 10-14 | 15-19 | 20-24 | 25-29 | 30-34 | 35-39 | 40-44 | 45-49 | 50-54 | 55-59 | 60-64 | 65-69 | 70-74 | 75-79 | 80-84 | 85+ |
| A | 0.4516 | 0.0490 | 0.0108 | 0.0052 | 0.0033 | 0.0042 | 0.0041 | 0.0055 | 0.0045 | 0.0043 | 0.0077 | 0.0076 | 0.0096 | 0.0145 | 0.0138 | 0.0150 | 0.0146 | 0.0083 | 0.0037 |
| A1 | 0.0009 | 0.0005 | 0.0007 | 0.0008 | 0.0009 | 0.0013 | 0.0010 | 0.0013 | 0.0007 | 0.0010 | 0.0030 | 0.0036 | 0.0054 | 0.0086 | 0.0081 | 0.0084 | 0.0068 | 0.0031 | 0.0012 |
| A2 | 0.2079 | 0.0463 | 0.0087 | 0.0036 | 0.0018 | 0.0018 | 0.0016 | 0.0018 | 0.0013 | 0.0010 | 0.0016 | 0.0014 | 0.0019 | 0.0034 | 0.0034 | 0.0049 | 0.0063 | 0.0044 | 0.0021 |
| A3 | 0.0005 | 0.0010 | 0.0006 | 0.0004 | 0.0002 | 0.0002 | 0.0002 | 0.0003 | 0.0003 | 0.0003 | 0.0003 | 0.0004 | 0.0003 | 0.0003 | 0.0002 | 0.0002 | 0.0001 | 0.0000 | 0.0000 |
| A4 | 0.0000 | 0.0000 | 0.0000 | 0.0000 | 0.0000 | 0.0000 | 0.0000 | 0.0000 | 0.0000 | 0.0000 | 0.0000 | 0.0000 | 0.0000 | 0.0000 | 0.0000 | 0.0000 | 0.0000 | 0.0000 | 0.0000 |
| A5 | 0.2369 | 0.0000 | 0.0000 | 0.0000 | 0.0000 | 0.0000 | 0.0000 | 0.0000 | 0.0000 | 0.0000 | 0.0000 | 0.0000 | 0.0000 | 0.0000 | 0.0000 | 0.0000 | 0.0000 | 0.0000 | 0.0000 |
| A6 | 0.0020 | 0.0002 | 0.0003 | 0.0002 | 0.0001 | 0.0002 | 0.0001 | 0.0001 | 0.0001 | 0.0001 | 0.0001 | 0.0001 | 0.0002 | 0.0002 | 0.0002 | 0.0003 | 0.0005 | 0.0004 | 0.0002 |
| A7 | 0.0034 | 0.0009 | 0.0005 | 0.0004 | 0.0003 | 0.0007 | 0.0012 | 0.0020 | 0.0021 | 0.0020 | 0.0026 | 0.0021 | 0.0018 | 0.0019 | 0.0019 | 0.0012 | 0.0008 | 0.0004 | 0.0002 |
| B | 0.1254 | 0.0255 | 0.0176 | 0.0166 | 0.0133 | 0.0238 | 0.0287 | 0.0461 | 0.0477 | 0.0356 | 0.0933 | 0.0725 | 0.1104 | 0.2332 | 0.2180 | 0.2749 | 0.2915 | 0.1350 | 0.0482 |
| B1 | 0.0019 | 0.0039 | 0.0059 | 0.0058 | 0.0048 | 0.0058 | 0.0080 | 0.0156 | 0.0197 | 0.0193 | 0.0426 | 0.0319 | 0.0348 | 0.0613 | 0.0441 | 0.0434 | 0.0340 | 0.0090 | 0.0023 |
| B2 | 0.0123 | 0.0038 | 0.0030 | 0.0030 | 0.0030 | 0.0064 | 0.0072 | 0.0117 | 0.0110 | 0.0033 | 0.0250 | 0.0136 | 0.0342 | 0.0927 | 0.0863 | 0.1083 | 0.1277 | 0.0528 | 0.0131 |
| B3 | 0.0000 | 0.0001 | 0.0005 | 0.0005 | 0.0007 | 0.0018 | 0.0022 | 0.0033 | 0.0036 | 0.0044 | 0.0099 | 0.0155 | 0.0271 | 0.0575 | 0.0714 | 0.1071 | 0.1137 | 0.0672 | 0.0313 |
| B4 | 0.0000 | 0.0005 | 0.0004 | 0.0004 | 0.0004 | 0.0012 | 0.0028 | 0.0052 | 0.0060 | 0.0048 | 0.0083 | 0.0063 | 0.0070 | 0.0092 | 0.0059 | 0.0051 | 0.0044 | 0.0016 | 0.0004 |
| B5 | -0.0008 | -0.0003 | 0.0008 | 0.0005 | 0.0005 | 0.0010 | 0.0013 | 0.0019 | 0.0018 | 0.0016 | 0.0028 | 0.0025 | 0.0034 | 0.0051 | 0.0047 | 0.0048 | 0.0048 | 0.0026 | 0.0012 |
| B6 | 0.0013 | 0.0012 | 0.0010 | 0.0013 | 0.0011 | 0.0015 | 0.0010 | 0.0012 | 0.0007 | 0.0003 | 0.0006 | 0.0003 | 0.0002 | 0.0002 | -0.0004 | -0.0007 | -0.0008 | -0.0011 | -0.0012 |
| B7 | 0.0000 | 0.0000 | 0.0000 | 0.0000 | 0.0013 | 0.0027 | 0.0027 | 0.0024 | 0.0014 | 0.0003 | 0.0004 | 0.0002 | 0.0005 | 0.0006 | 0.0003 | 0.0003 | 0.0002 | 0.0000 | 0.0001 |
| B8 | 0.0063 | 0.0032 | 0.0019 | 0.0020 | 0.0011 | 0.0027 | 0.0031 | 0.0044 | 0.0033 | 0.0016 | 0.0036 | 0.0021 | 0.0031 | 0.0065 | 0.0054 | 0.0063 | 0.0074 | 0.0030 | 0.0011 |
| B9 | 0.0000 | 0.0000 | 0.0001 | 0.0001 | 0.0000 | 0.0001 | 0.0001 | 0.0001 | 0.0001 | 0.0000 | 0.0001 | 0.0001 | 0.0001 | 0.0002 | 0.0002 | 0.0002 | 0.0002 | 0.0001 | 0.0000 |
| B10 | 0.1044 | 0.0130 | 0.0041 | 0.0030 | 0.0006 | 0.0007 | 0.0003 | 0.0004 | 0.0001 | 0.0000 | 0.0000 | 0.0000 | 0.0000 | 0.0000 | 0.0000 | 0.0000 | 0.0000 | 0.0000 | 0.0000 |
| C | 0.0258 | 0.0396 | 0.0466 | 0.0423 | 0.0112 | 0.0301 | 0.0296 | 0.0329 | 0.0134 | -0.0123 | 0.0044 | -0.0050 | -0.0036 | 0.0032 | 0.0020 | 0.0041 | 0.0055 | 0.0017 | -0.0001 |
| C1 | 0.0000 | 0.0000 | 0.0001 | 0.0000 | 0.0004 | 0.0020 | 0.0018 | 0.0018 | 0.0014 | 0.0007 | 0.0004 | 0.0002 | 0.0001 | 0.0001 | 0.0000 | 0.0000 | 0.0000 | 0.0000 | 0.0000 |
| C2 | 0.0020 | 0.0020 | 0.0020 | 0.0044 | 0.0062 | 0.0120 | 0.0121 | 0.0117 | 0.0078 | 0.0036 | 0.0054 | 0.0031 | 0.0025 | 0.0031 | 0.0018 | 0.0020 | 0.0018 | 0.0006 | 0.0001 |
| C3 | -0.0005 | 0.0007 | 0.0117 | 0.0094 | 0.0003 | 0.0123 | 0.0121 | 0.0163 | 0.0071 | -0.0082 | 0.0010 | -0.0048 | -0.0040 | -0.0007 | 0.0000 | 0.0013 | 0.0022 | 0.0008 | 0.0001 |
| C4 | 0.0243 | 0.0368 | 0.0329 | 0.0284 | 0.0043 | 0.0037 | 0.0035 | 0.0031 | -0.0030 | -0.0084 | -0.0024 | -0.0035 | -0.0022 | 0.0008 | 0.0002 | 0.0008 | 0.0014 | 0.0003 | -0.0004 |
| TE | 0.6028 | 0.1141 | 0.0750 | 0.0641 | 0.0279 | 0.0581 | 0.0624 | 0.0846 | 0.0656 | 0.0276 | 0.1053 | 0.0751 | 0.1164 | 0.2508 | 0.2338 | 0.2939 | 0.3116 | 0.1450 | 0.0518 |

*Note: TE, total effect to the life expectancy gap. Gray shadow represents negative effect to the life expectancy gap; Value of ‘0.0000’ represents |effect|<0.0001.*

**Table S6b** Number of years contributed by ages and causes to the life expectancy gap for female, China, 2002-2007.

| Causes of death | Age groups | | | | | | | | | | | | | | | | | | |
| --- | --- | --- | --- | --- | --- | --- | --- | --- | --- | --- | --- | --- | --- | --- | --- | --- | --- | --- | --- |
|  | <1 | 1-4 | 5-9 | 10-14 | 15-19 | 20-24 | 25-29 | 30-34 | 35-39 | 40-44 | 45-49 | 50-54 | 55-59 | 60-64 | 65-69 | 70-74 | 75-79 | 80-84 | 85+ |
| A | 0.4731 | 0.0574 | 0.0120 | 0.0058 | 0.0056 | 0.0090 | 0.0118 | 0.0095 | 0.0096 | 0.0064 | 0.0060 | 0.0067 | 0.0077 | 0.0116 | 0.0122 | 0.0132 | 0.0133 | 0.0121 | 0.0094 |
| A1 | 0.0010 | 0.0009 | 0.0005 | 0.0009 | 0.0013 | 0.0016 | 0.0021 | 0.0019 | 0.0026 | 0.0025 | 0.0028 | 0.0034 | 0.0042 | 0.0061 | 0.0059 | 0.0051 | 0.0044 | 0.0030 | 0.0017 |
| A2 | 0.2261 | 0.0525 | 0.0098 | 0.0037 | 0.0020 | 0.0018 | 0.0022 | 0.0017 | 0.0016 | 0.0013 | 0.0016 | 0.0017 | 0.0019 | 0.0036 | 0.0044 | 0.0062 | 0.0071 | 0.0077 | 0.0066 |
| A3 | 0.0007 | 0.0016 | 0.0009 | 0.0005 | 0.0002 | 0.0002 | 0.0002 | 0.0002 | 0.0003 | 0.0003 | 0.0003 | 0.0004 | 0.0004 | 0.0004 | 0.0003 | 0.0003 | 0.0002 | 0.0001 | 0.0000 |
| A4 | 0.0000 | 0.0000 | 0.0000 | 0.0002 | 0.0016 | 0.0046 | 0.0065 | 0.0048 | 0.0038 | 0.0012 | 0.0003 | 0.0001 | 0.0000 | 0.0000 | 0.0000 | 0.0000 | 0.0000 | 0.0000 | 0.0000 |
| A5 | 0.2224 | 0.0005 | 0.0000 | 0.0000 | 0.0000 | 0.0000 | 0.0000 | 0.0000 | 0.0000 | 0.0000 | 0.0000 | 0.0000 | 0.0000 | 0.0000 | 0.0000 | 0.0000 | 0.0000 | 0.0000 | 0.0000 |
| A6 | 0.0192 | 0.0005 | 0.0003 | 0.0002 | 0.0001 | 0.0002 | 0.0002 | 0.0002 | 0.0002 | 0.0002 | 0.0001 | 0.0001 | 0.0002 | 0.0003 | 0.0004 | 0.0006 | 0.0008 | 0.0010 | 0.0008 |
| A7 | 0.0037 | 0.0013 | 0.0005 | 0.0003 | 0.0004 | 0.0006 | 0.0006 | 0.0007 | 0.0010 | 0.0010 | 0.0010 | 0.0011 | 0.0011 | 0.0012 | 0.0012 | 0.0010 | 0.0008 | 0.0004 | 0.0002 |
| B | 0.1260 | 0.0388 | 0.0181 | 0.0160 | 0.0155 | 0.0210 | 0.0387 | 0.0417 | 0.0685 | 0.0690 | 0.0930 | 0.0898 | 0.0941 | 0.2081 | 0.2278 | 0.2510 | 0.2624 | 0.1755 | 0.1022 |
| B1 | 0.0060 | 0.0092 | 0.0063 | 0.0054 | 0.0047 | 0.0054 | 0.0116 | 0.0154 | 0.0265 | 0.0264 | 0.0337 | 0.0261 | 0.0221 | 0.0469 | 0.0410 | 0.0355 | 0.0269 | 0.0101 | 0.0045 |
| B2 | 0.0130 | 0.0053 | 0.0031 | 0.0031 | 0.0034 | 0.0061 | 0.0116 | 0.0118 | 0.0219 | 0.0245 | 0.0356 | 0.0365 | 0.0381 | 0.0918 | 0.1003 | 0.1015 | 0.1099 | 0.0684 | 0.0266 |
| B3 | 0.0000 | 0.0017 | 0.0006 | 0.0005 | 0.0010 | 0.0013 | 0.0029 | 0.0035 | 0.0052 | 0.0066 | 0.0099 | 0.0151 | 0.0223 | 0.0477 | 0.0655 | 0.0963 | 0.1087 | 0.0873 | 0.0648 |
| B4 | 0.0000 | 0.0008 | 0.0004 | 0.0004 | 0.0004 | 0.0007 | 0.0013 | 0.0017 | 0.0029 | 0.0032 | 0.0044 | 0.0044 | 0.0044 | 0.0061 | 0.0058 | 0.0048 | 0.0039 | 0.0023 | 0.0011 |
| B5 | 0.0007 | 0.0000 | 0.0008 | 0.0006 | 0.0004 | 0.0007 | 0.0013 | 0.0013 | 0.0016 | 0.0014 | 0.0018 | 0.0021 | 0.0026 | 0.0043 | 0.0047 | 0.0057 | 0.0056 | 0.0040 | 0.0029 |
| B6 | 0.0019 | 0.0014 | 0.0010 | 0.0010 | 0.0009 | 0.0010 | 0.0015 | 0.0012 | 0.0012 | 0.0007 | 0.0006 | 0.0005 | 0.0001 | 0.0000 | -0.0004 | -0.0009 | -0.0008 | -0.0003 | -0.0002 |
| B7 | 0.0000 | 0.0000 | 0.0000 | 0.0000 | 0.0011 | 0.0016 | 0.0023 | 0.0018 | 0.0016 | 0.0011 | 0.0008 | 0.0005 | 0.0004 | 0.0004 | 0.0004 | 0.0003 | 0.0004 | 0.0001 | 0.0001 |
| B8 | 0.0071 | 0.0042 | 0.0023 | 0.0021 | 0.0019 | 0.0025 | 0.0043 | 0.0040 | 0.0062 | 0.0046 | 0.0056 | 0.0043 | 0.0039 | 0.0103 | 0.0100 | 0.0074 | 0.0073 | 0.0034 | 0.0023 |
| B9 | 0.0000 | 0.0000 | 0.0002 | 0.0004 | 0.0006 | 0.0006 | 0.0009 | 0.0006 | 0.0008 | 0.0005 | 0.0004 | 0.0003 | 0.0002 | 0.0005 | 0.0005 | 0.0004 | 0.0004 | 0.0002 | 0.0001 |
| B10 | 0.0973 | 0.0162 | 0.0034 | 0.0025 | 0.0010 | 0.0008 | 0.0011 | 0.0005 | 0.0004 | 0.0002 | 0.0001 | 0.0000 | 0.0000 | 0.0000 | 0.0000 | 0.0000 | 0.0001 | 0.0001 | 0.0001 |
| C | 0.0251 | 0.0425 | 0.0287 | 0.0212 | 0.0150 | 0.0203 | 0.0315 | 0.0255 | 0.0254 | 0.0125 | 0.0113 | 0.0042 | 0.0007 | 0.0048 | 0.0040 | 0.0041 | 0.0038 | 0.0016 | -0.0013 |
| C1 | 0.0000 | 0.0000 | 0.0000 | 0.0000 | 0.0000 | 0.0000 | 0.0000 | 0.0000 | 0.0000 | 0.0000 | 0.0000 | 0.0000 | 0.0000 | 0.0000 | 0.0000 | 0.0000 | 0.0000 | 0.0000 | 0.0000 |
| C2 | 0.0038 | 0.0025 | 0.0020 | 0.0044 | 0.0079 | 0.0135 | 0.0189 | 0.0150 | 0.0130 | 0.0071 | 0.0062 | 0.0036 | 0.0019 | 0.0024 | 0.0018 | 0.0020 | 0.0018 | 0.0010 | 0.0003 |
| C3 | 0.0043 | 0.0079 | 0.0098 | 0.0056 | 0.0037 | 0.0039 | 0.0081 | 0.0069 | 0.0089 | 0.0038 | 0.0033 | 0.0001 | -0.0010 | 0.0012 | 0.0012 | 0.0013 | 0.0012 | 0.0007 | 0.0002 |
| C4 | 0.0170 | 0.0320 | 0.0169 | 0.0111 | 0.0034 | 0.0028 | 0.0046 | 0.0036 | 0.0035 | 0.0015 | 0.0018 | 0.0004 | -0.0002 | 0.0012 | 0.0011 | 0.0008 | 0.0008 | -0.0001 | -0.0018 |
| TE | 0.6243 | 0.1386 | 0.0588 | 0.0430 | 0.0360 | 0.0502 | 0.0821 | 0.0768 | 0.1035 | 0.0879 | 0.1103 | 0.1007 | 0.1025 | 0.2245 | 0.2440 | 0.2682 | 0.2796 | 0.1893 | 0.1102 |

*Note: TE, total effect to the life expectancy gap. Gray shadow represents negative effect to the life expectancy gap; Value of ‘0.0000’ represents |effect|<0.0001.*

**Table S7a** Number of years contributed by ages and causes to the life expectancy gap for male, China, 2007-2016.

| Causes of death | Age groups | | | | | | | | | | | | | | | | | | |
| --- | --- | --- | --- | --- | --- | --- | --- | --- | --- | --- | --- | --- | --- | --- | --- | --- | --- | --- | --- |
|  | <1 | 1-4 | 5-9 | 10-14 | 15-19 | 20-24 | 25-29 | 30-34 | 35-39 | 40-44 | 45-49 | 50-54 | 55-59 | 60-64 | 65-69 | 70-74 | 75-79 | 80-84 | 85+ |
| A | 0.4350 | 0.0350 | 0.0079 | 0.0026 | 0.0031 | 0.0042 | 0.0030 | 0.0025 | 0.0032 | 0.0047 | 0.0047 | 0.0076 | 0.0097 | 0.0081 | 0.0091 | 0.0113 | 0.0095 | 0.0076 | 0.0054 |
| A1 | 0.0005 | 0.0003 | 0.0002 | 0.0000 | 0.0010 | 0.0020 | 0.0013 | 0.0002 | 0.0001 | 0.0011 | 0.0018 | 0.0039 | 0.0056 | 0.0051 | 0.0054 | 0.0062 | 0.0045 | 0.0028 | 0.0015 |
| A2 | 0.1373 | 0.0326 | 0.0061 | 0.0016 | 0.0016 | 0.0015 | 0.0010 | 0.0011 | 0.0015 | 0.0018 | 0.0013 | 0.0018 | 0.0023 | 0.0017 | 0.0024 | 0.0042 | 0.0042 | 0.0044 | 0.0038 |
| A3 | 0.0003 | 0.0009 | 0.0013 | 0.0008 | 0.0003 | 0.0003 | 0.0002 | 0.0003 | 0.0004 | 0.0004 | 0.0004 | 0.0004 | 0.0005 | 0.0004 | 0.0003 | 0.0003 | 0.0002 | 0.0001 | 0.0000 |
| A4 | 0.0000 | 0.0000 | 0.0000 | 0.0000 | 0.0000 | 0.0000 | 0.0000 | 0.0000 | 0.0000 | 0.0000 | 0.0000 | 0.0000 | 0.0000 | 0.0000 | 0.0000 | 0.0000 | 0.0000 | 0.0000 | 0.0000 |
| A5 | 0.2943 | 0.0010 | 0.0000 | 0.0000 | 0.0000 | 0.0000 | 0.0000 | 0.0000 | 0.0000 | 0.0000 | 0.0000 | 0.0000 | 0.0000 | 0.0000 | 0.0000 | 0.0000 | 0.0000 | 0.0000 | 0.0000 |
| A6 | 0.0013 | 0.0001 | 0.0001 | 0.0000 | 0.0001 | 0.0001 | 0.0000 | 0.0000 | 0.0001 | 0.0001 | 0.0000 | 0.0001 | 0.0001 | 0.0000 | 0.0000 | 0.0000 | 0.0001 | 0.0001 | -0.0001 |
| A7 | 0.0013 | 0.0002 | 0.0002 | 0.0001 | 0.0002 | 0.0004 | 0.0005 | 0.0008 | 0.0011 | 0.0013 | 0.0012 | 0.0014 | 0.0013 | 0.0008 | 0.0009 | 0.0006 | 0.0004 | 0.0002 | 0.0001 |
| B | 0.1163 | 0.0276 | 0.0136 | 0.0086 | 0.0120 | 0.0174 | 0.0099 | 0.0241 | 0.0595 | 0.1045 | 0.0707 | 0.1275 | 0.1567 | 0.0742 | 0.1240 | 0.1933 | 0.1478 | 0.0994 | 0.0466 |
| B1 | 0.0006 | 0.0034 | 0.0035 | 0.0032 | 0.0042 | 0.0042 | 0.0029 | 0.0085 | 0.0230 | 0.0418 | 0.0362 | 0.0587 | 0.0641 | 0.0201 | 0.0290 | 0.0434 | 0.0288 | 0.0104 | 0.0036 |
| B2 | 0.0138 | 0.0050 | 0.0027 | 0.0018 | 0.0027 | 0.0043 | -0.0009 | 0.0038 | 0.0170 | 0.0356 | 0.0152 | 0.0372 | 0.0517 | 0.0195 | 0.0425 | 0.0666 | 0.0451 | 0.0316 | 0.0092 |
| B3 | 0.0000 | 0.0001 | 0.0004 | 0.0002 | 0.0006 | 0.0012 | 0.0013 | 0.0019 | 0.0033 | 0.0060 | 0.0064 | 0.0142 | 0.0228 | 0.0290 | 0.0461 | 0.0736 | 0.0679 | 0.0537 | 0.0326 |
| B4 | 0.0000 | 0.0004 | 0.0003 | 0.0002 | 0.0004 | 0.0011 | 0.0017 | 0.0034 | 0.0065 | 0.0097 | 0.0062 | 0.0081 | 0.0081 | 0.0029 | 0.0029 | 0.0035 | 0.0022 | 0.0012 | 0.0005 |
| B5 | -0.0007 | 0.0006 | 0.0007 | 0.0003 | 0.0005 | 0.0009 | 0.0009 | 0.0013 | 0.0019 | 0.0025 | 0.0022 | 0.0032 | 0.0038 | 0.0029 | 0.0035 | 0.0046 | 0.0037 | 0.0028 | 0.0017 |
| B6 | 0.0005 | 0.0009 | 0.0007 | 0.0004 | 0.0006 | 0.0008 | 0.0004 | 0.0007 | 0.0009 | 0.0008 | 0.0003 | 0.0006 | 0.0005 | -0.0002 | -0.0004 | -0.0004 | -0.0008 | -0.0003 | -0.0008 |
| B7 | 0.0000 | 0.0000 | 0.0000 | 0.0000 | 0.0004 | 0.0011 | 0.0012 | 0.0013 | 0.0016 | 0.0019 | 0.0012 | 0.0014 | 0.0010 | -0.0001 | -0.0002 | -0.0002 | -0.0002 | -0.0002 | -0.0001 |
| B8 | 0.0035 | 0.0029 | 0.0016 | 0.0010 | 0.0015 | 0.0026 | 0.0022 | 0.0029 | 0.0048 | 0.0058 | 0.0028 | 0.0039 | 0.0046 | 0.0001 | 0.0004 | 0.0019 | 0.0009 | 0.0000 | -0.0003 |
| B9 | 0.0000 | 0.0000 | 0.0001 | 0.0000 | 0.0001 | 0.0001 | 0.0001 | 0.0001 | 0.0001 | 0.0002 | 0.0001 | 0.0002 | 0.0002 | 0.0001 | 0.0001 | 0.0001 | 0.0001 | 0.0001 | 0.0000 |
| B10 | 0.0985 | 0.0142 | 0.0036 | 0.0016 | 0.0011 | 0.0009 | 0.0002 | 0.0003 | 0.0004 | 0.0003 | 0.0001 | 0.0001 | 0.0001 | 0.0000 | 0.0001 | 0.0001 | 0.0001 | 0.0001 | 0.0001 |
| C | 0.0207 | 0.0525 | 0.0508 | 0.0265 | 0.0362 | 0.0594 | 0.0383 | 0.0498 | 0.0659 | 0.0592 | 0.0239 | 0.0252 | 0.0178 | 0.0016 | 0.0039 | 0.0074 | 0.0057 | 0.0039 | 0.0017 |
| C1 | 0.0000 | 0.0000 | 0.0000 | 0.0000 | 0.0001 | 0.0007 | 0.0004 | 0.0004 | 0.0004 | 0.0003 | 0.0001 | 0.0001 | 0.0000 | 0.0000 | 0.0000 | 0.0000 | 0.0000 | 0.0000 | 0.0000 |
| C2 | 0.0013 | 0.0015 | 0.0014 | 0.0024 | 0.0063 | 0.0096 | 0.0067 | 0.0077 | 0.0097 | 0.0091 | 0.0056 | 0.0065 | 0.0058 | 0.0033 | 0.0034 | 0.0039 | 0.0030 | 0.0019 | 0.0008 |
| C3 | -0.0009 | 0.0070 | 0.0156 | 0.0056 | 0.0179 | 0.0342 | 0.0218 | 0.0281 | 0.0366 | 0.0332 | 0.0118 | 0.0114 | 0.0071 | -0.0013 | 0.0002 | 0.0017 | 0.0015 | 0.0010 | 0.0005 |
| C4 | 0.0204 | 0.0441 | 0.0338 | 0.0185 | 0.0119 | 0.0149 | 0.0093 | 0.0136 | 0.0193 | 0.0166 | 0.0064 | 0.0072 | 0.0050 | -0.0003 | 0.0003 | 0.0018 | 0.0012 | 0.0010 | 0.0004 |
| TE | 0.5720 | 0.1151 | 0.0724 | 0.0378 | 0.0514 | 0.0809 | 0.0512 | 0.0764 | 0.1287 | 0.1683 | 0.0993 | 0.1604 | 0.1843 | 0.0839 | 0.1369 | 0.2120 | 0.1631 | 0.1109 | 0.0536 |

*Note: TE, total effect to the life expectancy gap. Gray shadow represents negative effect to the life expectancy gap; Value of ‘0.0000’ represents |effect|<0.0001.*

**Table S7b** Number of years contributed by ages and causes to the life expectancy gap for female, China, 2007-2016.

| Causes of death | Age groups | | | | | | | | | | | | | | | | | | |
| --- | --- | --- | --- | --- | --- | --- | --- | --- | --- | --- | --- | --- | --- | --- | --- | --- | --- | --- | --- |
|  | <1 | 1-4 | 5-9 | 10-14 | 15-19 | 20-24 | 25-29 | 30-34 | 35-39 | 40-44 | 45-49 | 50-54 | 55-59 | 60-64 | 65-69 | 70-74 | 75-79 | 80-84 | 85+ |
| A | 0.4165 | 0.0336 | 0.0049 | 0.0020 | 0.0046 | 0.0084 | 0.0065 | 0.0056 | 0.0057 | 0.0051 | 0.0040 | 0.0062 | 0.0081 | 0.0084 | 0.0106 | 0.0139 | 0.0133 | 0.0144 | 0.0169 |
| A1 | 0.0004 | 0.0004 | -0.0001 | 0.0000 | 0.0012 | 0.0022 | 0.0011 | 0.0004 | 0.0014 | 0.0021 | 0.0019 | 0.0030 | 0.0040 | 0.0040 | 0.0043 | 0.0044 | 0.0032 | 0.0022 | 0.0015 |
| A2 | 0.1270 | 0.0300 | 0.0040 | 0.0014 | 0.0017 | 0.0017 | 0.0011 | 0.0011 | 0.0011 | 0.0012 | 0.0011 | 0.0018 | 0.0026 | 0.0031 | 0.0049 | 0.0079 | 0.0087 | 0.0110 | 0.0142 |
| A3 | 0.0003 | 0.0011 | 0.0007 | 0.0004 | 0.0002 | 0.0001 | 0.0001 | 0.0002 | 0.0002 | 0.0003 | 0.0003 | 0.0004 | 0.0004 | 0.0004 | 0.0004 | 0.0004 | 0.0002 | 0.0001 | 0.0001 |
| A4 | 0.0000 | 0.0000 | 0.0000 | 0.0001 | 0.0012 | 0.0039 | 0.0039 | 0.0036 | 0.0025 | 0.0009 | 0.0002 | 0.0001 | 0.0000 | 0.0000 | 0.0000 | 0.0000 | 0.0000 | 0.0000 | 0.0000 |
| A5 | 0.2778 | 0.0009 | 0.0000 | 0.0000 | 0.0000 | 0.0000 | 0.0000 | 0.0000 | 0.0000 | 0.0000 | 0.0000 | 0.0000 | 0.0000 | 0.0000 | 0.0000 | 0.0000 | 0.0000 | 0.0000 | 0.0000 |
| A6 | 0.0084 | 0.0003 | 0.0001 | 0.0000 | 0.0001 | 0.0001 | 0.0001 | 0.0001 | 0.0001 | 0.0001 | 0.0001 | 0.0001 | 0.0001 | 0.0002 | 0.0002 | 0.0004 | 0.0005 | 0.0007 | 0.0009 |
| A7 | 0.0026 | 0.0010 | 0.0002 | 0.0001 | 0.0002 | 0.0003 | 0.0002 | 0.0003 | 0.0004 | 0.0005 | 0.0004 | 0.0008 | 0.0010 | 0.0008 | 0.0009 | 0.0009 | 0.0006 | 0.0004 | 0.0002 |
| B | 0.1130 | 0.0369 | 0.0031 | 0.0047 | 0.0171 | 0.0203 | 0.0162 | 0.0269 | 0.0513 | 0.0840 | 0.0768 | 0.1488 | 0.2224 | 0.2166 | 0.3209 | 0.4287 | 0.3621 | 0.2845 | 0.2108 |
| B1 | 0.0028 | 0.0066 | 0.0003 | 0.0012 | 0.0050 | 0.0051 | 0.0044 | 0.0095 | 0.0203 | 0.0329 | 0.0287 | 0.0498 | 0.0675 | 0.0532 | 0.0742 | 0.0831 | 0.0519 | 0.0262 | 0.0137 |
| B2 | 0.0129 | 0.0055 | 0.0010 | 0.0012 | 0.0042 | 0.0062 | 0.0052 | 0.0087 | 0.0179 | 0.0326 | 0.0307 | 0.0660 | 0.1023 | 0.1031 | 0.1542 | 0.2064 | 0.1790 | 0.1393 | 0.0941 |
| B3 | 0.0000 | 0.0011 | 0.0002 | 0.0002 | 0.0008 | 0.0011 | 0.0013 | 0.0019 | 0.0030 | 0.0050 | 0.0065 | 0.0140 | 0.0255 | 0.0375 | 0.0635 | 0.1068 | 0.1105 | 0.1053 | 0.0941 |
| B4 | 0.0000 | 0.0005 | 0.0001 | 0.0001 | 0.0004 | 0.0006 | 0.0006 | 0.0011 | 0.0019 | 0.0029 | 0.0028 | 0.0052 | 0.0072 | 0.0062 | 0.0069 | 0.0068 | 0.0044 | 0.0028 | 0.0016 |
| B5 | 0.0005 | 0.0010 | 0.0003 | 0.0003 | 0.0005 | 0.0007 | 0.0007 | 0.0008 | 0.0010 | 0.0014 | 0.0014 | 0.0024 | 0.0037 | 0.0039 | 0.0055 | 0.0076 | 0.0072 | 0.0061 | 0.0054 |
| B6 | 0.0008 | 0.0010 | 0.0002 | 0.0002 | 0.0009 | 0.0008 | 0.0005 | 0.0006 | 0.0007 | 0.0007 | 0.0005 | 0.0007 | 0.0005 | -0.0001 | -0.0002 | -0.0006 | -0.0025 | -0.0006 | -0.0006 |
| B7 | 0.0000 | 0.0000 | 0.0000 | 0.0000 | 0.0005 | 0.0006 | 0.0002 | 0.0004 | 0.0005 | 0.0005 | 0.0001 | 0.0002 | 0.0003 | -0.0001 | 0.0000 | -0.0001 | -0.0002 | -0.0001 | -0.0001 |
| B8 | 0.0033 | 0.0031 | 0.0004 | 0.0006 | 0.0022 | 0.0029 | 0.0022 | 0.0030 | 0.0049 | 0.0069 | 0.0057 | 0.0097 | 0.0146 | 0.0124 | 0.0160 | 0.0177 | 0.0110 | 0.0049 | 0.0019 |
| B9 | 0.0000 | 0.0000 | 0.0000 | 0.0001 | 0.0009 | 0.0008 | 0.0004 | 0.0004 | 0.0006 | 0.0007 | 0.0004 | 0.0006 | 0.0006 | 0.0004 | 0.0006 | 0.0008 | 0.0006 | 0.0003 | 0.0002 |
| B10 | 0.0927 | 0.0181 | 0.0006 | 0.0009 | 0.0017 | 0.0014 | 0.0006 | 0.0004 | 0.0004 | 0.0003 | 0.0002 | 0.0002 | 0.0002 | 0.0001 | 0.0002 | 0.0002 | 0.0002 | 0.0003 | 0.0005 |
| C | 0.0203 | 0.0499 | 0.0088 | 0.0094 | 0.0250 | 0.0303 | 0.0221 | 0.0252 | 0.0286 | 0.0272 | 0.0166 | 0.0218 | 0.0216 | 0.0143 | 0.0171 | 0.0191 | 0.0156 | 0.0125 | 0.0091 |
| C1 | 0.0000 | 0.0000 | 0.0000 | 0.0000 | 0.0000 | 0.0000 | 0.0000 | 0.0000 | 0.0000 | 0.0000 | 0.0000 | 0.0000 | 0.0000 | 0.0000 | 0.0000 | 0.0000 | 0.0000 | 0.0000 | 0.0000 |
| C2 | 0.0023 | 0.0019 | 0.0005 | 0.0021 | 0.0090 | 0.0132 | 0.0118 | 0.0125 | 0.0131 | 0.0114 | 0.0087 | 0.0107 | 0.0100 | 0.0076 | 0.0086 | 0.0091 | 0.0072 | 0.0050 | 0.0027 |
| C3 | 0.0025 | 0.0124 | 0.0027 | 0.0026 | 0.0104 | 0.0117 | 0.0067 | 0.0085 | 0.0108 | 0.0110 | 0.0052 | 0.0071 | 0.0071 | 0.0038 | 0.0046 | 0.0046 | 0.0030 | 0.0016 | 0.0010 |
| C4 | 0.0154 | 0.0357 | 0.0056 | 0.0047 | 0.0056 | 0.0053 | 0.0036 | 0.0042 | 0.0047 | 0.0047 | 0.0027 | 0.0041 | 0.0045 | 0.0030 | 0.0039 | 0.0054 | 0.0054 | 0.0059 | 0.0055 |
| TE | 0.5498 | 0.1204 | 0.0168 | 0.0161 | 0.0468 | 0.0590 | 0.0448 | 0.0577 | 0.0857 | 0.1163 | 0.0974 | 0.1768 | 0.2522 | 0.2393 | 0.3486 | 0.4617 | 0.3910 | 0.3113 | 0.2369 |

*Note: TE, total effect to the life expectancy gap. Gray shadow represents negative effect to the life expectancy gap; Value of ‘0.0000’ represents |effect|<0.0001.*
